# Heparins are potent inhibitors of ectonucleotide pyrophosphatase/phospho-diesterase-1 (NPP1) – a promising target for the immunotherapy of cancer

**DOI:** 10.3389/fimmu.2023.1173634

**Published:** 2023-08-29

**Authors:** Vittoria Lopez, H. J. Maximilian Schuh, Salahuddin Mirza, Victoria J. Vaaßen, Michael S. Schmidt, Katharina Sylvester, Riham M. Idris, Christian Renn, Laura Schäkel, Julie Pelletier, Jean Sévigny, Annamaria Naggi, Björn Scheffler, Sang-Yong Lee, Gerd Bendas, Christa E. Müller

**Affiliations:** ^1^ Pharmaceutical Institute, Pharmaceutical and Medicinal Chemistry, University of Bonn, Bonn, Germany; ^2^ PharmaCenter Bonn, University of Bonn, Bonn, Germany; ^3^ Pharmaceutical Institute, Pharmaceutical and Cell Biological Chemistry, University of Bonn, Bonn, Germany; ^4^ Centre de Recherche du CHU de Québec-Université Laval, Québec, QC, Canada; ^5^ Départment de Microbiologie-Infectiologie et d’Immunologie, Faculté de Médecine, Université Laval, Quebec, QC, Canada; ^6^ Institute for Chemical and Biochemical Research “G. Ronzoni”, Milan, Italy; ^7^ DKFZ Division Translational Neurooncology at the West German Cancer Center (WTZ), DKTK Partner site, University Hospital Essen and German Cancer Research Center, Heidelberg, Germany

**Keywords:** adenosine, ectonucleotidase inhibitors, immuno-oncology, NPP1, U87 glioblastoma cells, heparin

## Abstract

**Introduction:**

Heparins, naturally occurring glycosaminoglycans, are widely used for thrombosis prevention. Upon application as anticoagulants in cancer patients, heparins were found to possess additional antitumor activities. Ectonucleotidases have recently been proposed as novel targets for cancer immunotherapy.

**Methods and results:**

In the present study, we discovered that heparin and its derivatives act as potent, selective, allosteric inhibitors of the poorly investigated ectonucleotidase NPP1 (nucleotide pyrophosphatase/phosphodiesterase-1, CD203a). Structure-activity relationships indicated that NPP1 inhibition could be separated from the compounds’ antithrombotic effect. Moreover, unfractionated heparin (UFH) and different low molecular weight heparins (LMWHs) inhibited extracellular adenosine production by the NPP1-expressing glioma cell line U87 at therapeutically relevant concentrations. As a consequence, heparins inhibited the ability of U87 cell supernatants to induce CD4+ T cell differentiation into immunosuppressive Treg cells.

**Discussion:**

NPP1 inhibition likely contributes to the anti-cancer effects of heparins, and their specific optimization may lead to improved therapeutics for the immunotherapy of cancer.

## 1 Introduction

Heparin is a heterogeneous mixture of linear, sulfated glycosaminoglycan polymers naturally occurring in the body. It prevents the formation of blood clots, and has therefore been established since decades as a subcutaneously applied anticoagulant drug. While other, orally available anticoagulants are becoming increasingly important, heparin has retained its outstanding role as a drug of choice in oncology for the prophylaxis and treatment of cancer-associated thrombosis (1–5). Several clinical studies suggest that heparins have a survival benefit for cancer patients (6). Moreover, heparin’s potential to inhibit cancer proliferation and metastasis has been demonstrated in various animal models (7, 8). Several potential targets for heparin have been postulated to be responsible for its antimetastatic activity. Apart from its anticoagulant activity, which is mediated by antithrombin, the inhibition of heparanase and thus tumor cell invasiveness (9), and the attenuation of adhesion receptor functionality, e.g. the suppression of P- and L-selectin and of selected integrins, are discussed as mechanisms of action (10). However, the precise mechanism by which heparins exert their anti-cancer effects and enhance cancer patient survival is still unknown.

**Figure 1:**
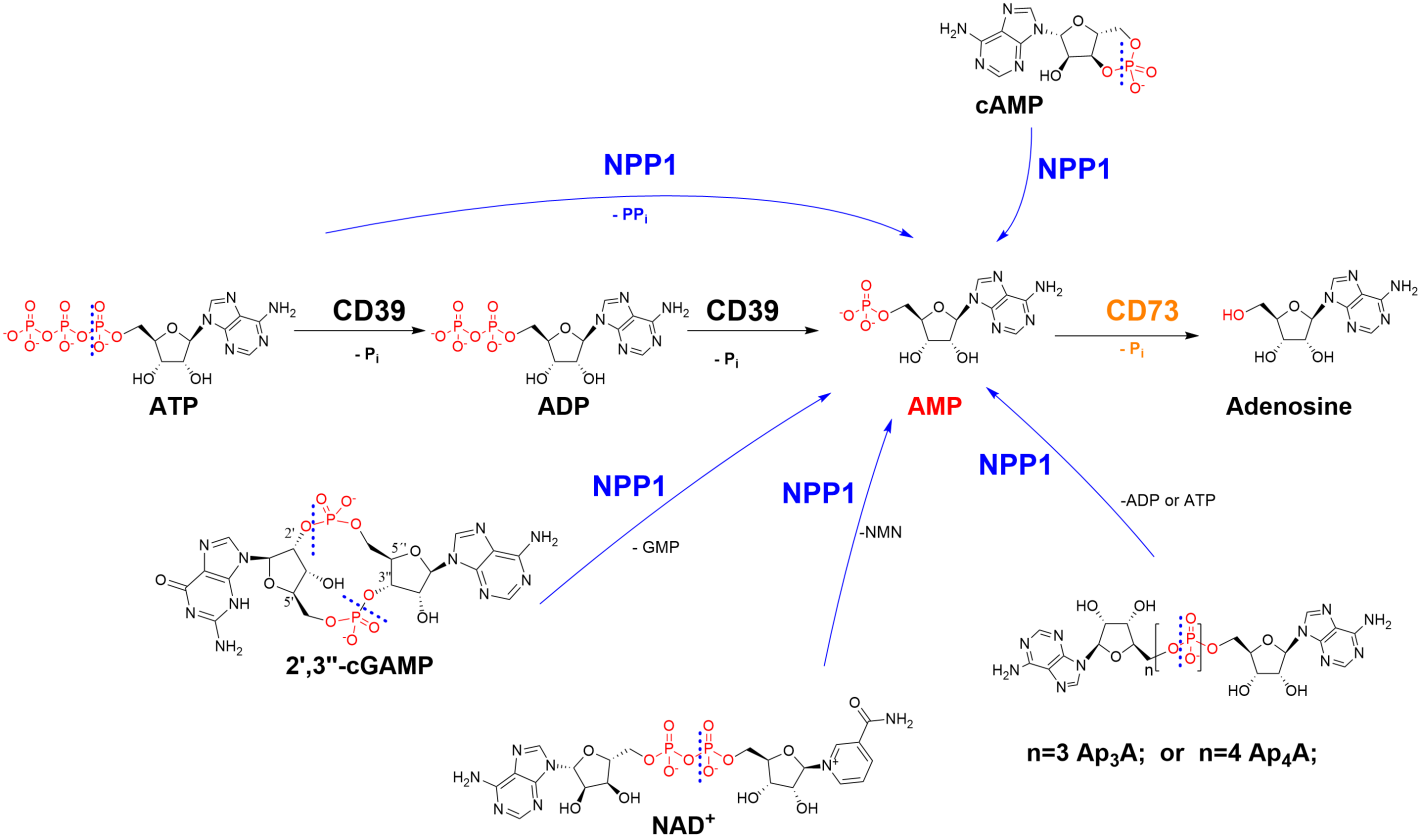
Ectonucleotidases present in cell membranes catalyze the hydrolysis of extracellular nucleotides finally yielding cancer-promoting, immunosuppressive adenosine. NPP1 (CD203a): ecto-nucleotide pyrophosphatase/phosphodiesterase-1; CD39: ecto-nucleoside triphosphate diphosphohydrolase-1 (NTPDase1); CD73: ecto-5’-nucleotidase. CD, cluster of differentiation; NMN, nicotineamide mononucleotide; P_i_, inorganic phosphate; PP_i_, diphosphate (pyrophosphate). The cleavage sites of NPP1 are indicated by dashed lines.

We recently observed that sulfopolysaccharides isolated from sea algae can inhibit the ATP-hydrolyzing ectonucleotidases NPP1 (CD203a; CD, cluster of differentiation) and NTPDase1 (CD39) (11), enzymes whose important role in cancer proliferation, angiogenesis, metastasis, and immune escape is increasingly recognized (12, 13). Ectonucleotidases are expressed on cancer cells, catalyzing the hydrolysis of ATP released by the cells due to hypoxic conditions and cell death, producing cancer-promoting, immunosuppressive adenosine (see **Figure 1**) (12, 13). These surface-bound ectonucleotidases include ectonucleoside triphosphate diphosphohydrolases (NTPDases, EC 3.6.1.5), ectonucleotide pyrophosphatases/phosphodiesterases (NPPs, EC 3.6.1.9), and ecto-5′-nucleotidase (CD73, EC 3.1.3.5) (12, 13). NTPDases convert nucleoside 5′-triphosphates (NTPs) to nucleoside 5′-diphosphates (NDPs) and further to nucleoside 5′-monophosphates (NMPs) (14). The NPP subfamily of ectonucleotidases degrades NTPs directly to NMPs, releasing diphosphate (pyrophosphate, PP_i_) (12, 14). CD73 further hydrolyzes NMPs to nucleosides, primarily AMP to adenosine (12, 14). The main substrate of human NPP1, or CD203a, previously known as PC-1 (plasma cell membrane glycoprotein-1), is ATP (15). In addition, it hydrolyzes further nucleotides, e.g. nicotinamide adenine dinucleotide (NAD^+^), diadenosine polyphosphates and cyclic dinucleotides such as cyclic guanosine-(2′,5′)-monophosphate-adenosine-(3′,5′′)-monophosphate (2′,3′-cGAMP) (see **Figure 1**). This cyclic dinucleotide is a natural agonist of the stimulator of interferon genes (STING), which is crucial for the induction of a type I interferon response by the innate immune system (15–17). Inhibiting the hydrolysis of immunostimulatory ATP and cGAMP and the subsequent production of immunosuppressive, cancer-promoting adenosine has been proposed as a novel strategy to treat cancer and infections (18).

In the present study, we investigated the potential of heparin and heparin-derived sulfopolysaccharides to inhibit ectonucleotidases. In fact, we discovered that heparin and its derivatives are potent and selective allosteric inhibitors of NPP1. This mechanism of action may contribute to the anti-cancer effects observed for heparins. The present results and the observed structure-activity relationships provide the basis for the future development of more potent NPP1 inhibitors lacking the associated anti-thrombotic activity.

## 2 Materials and methods

### 2.1 Chemicals

**Figure 2:**
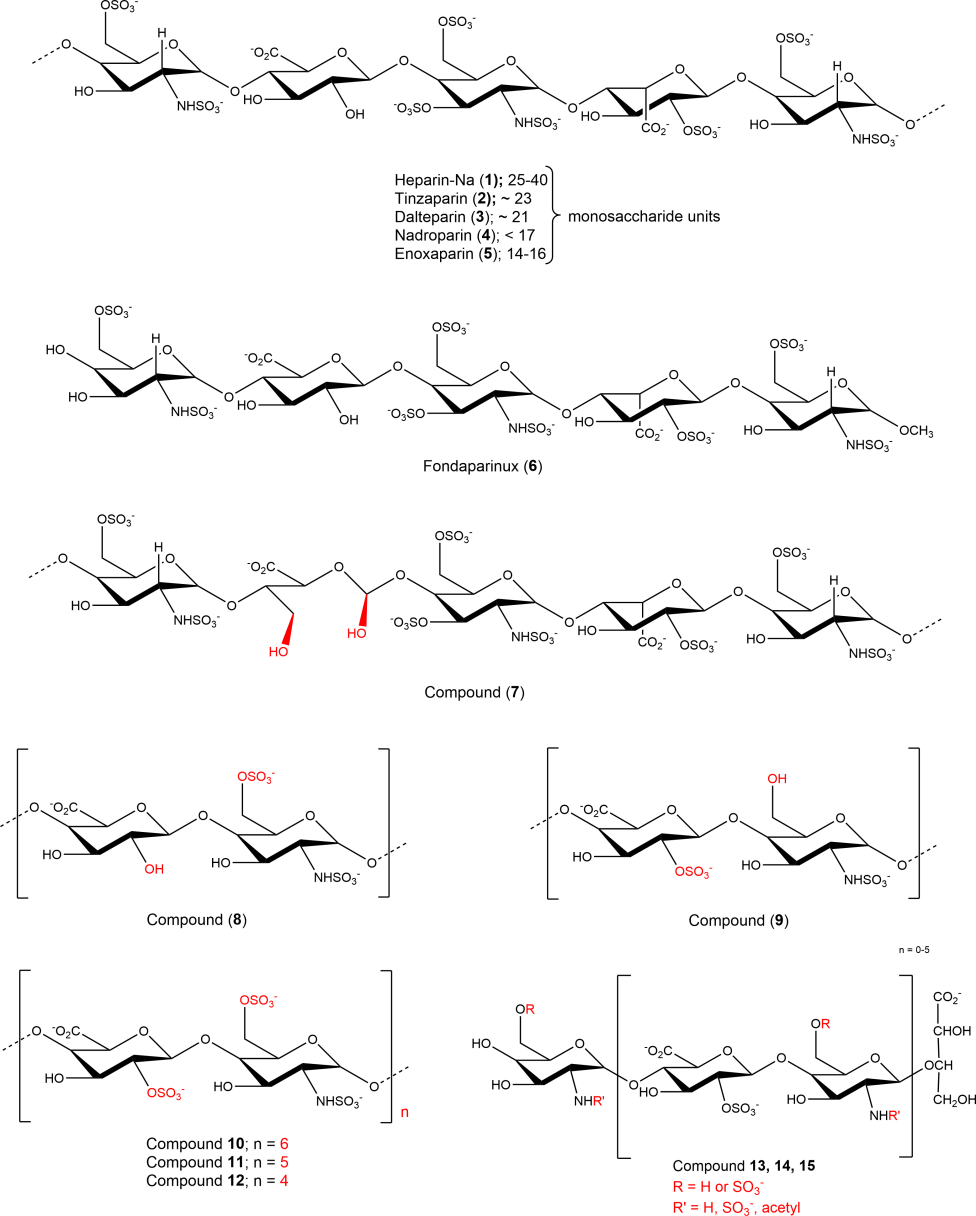
Structures of investigated sulfopolysaccharides. For details see **Table 1**.

Nucleotides (AMP, ADP, ATP, cAMP), nucleosides (adenosine, inosine, uridine), dipyridamole (DP), EHNA (erythro-9-(2-hydroxy-3-nonyl)adenine), disodium hydrogen phosphate, and sodium dodecyl sulfate were purchased from Sigma-Aldrich^®^, Merck KGaA, Darmstadt, Germany. Sodium chloride, potassium chloride, potassium dihydrogen phosphate, sodium hydrogencarbonate, D-glucose, HEPES (*N*-[2-hydroxyethyl]-piperazine-*N*’-[2-ethanesulfonic acid]), calcium chloride, and magnesium sulfate were purchased from PAN Biotech GmbH (Aidenbach, Germany). Dulbecco’s Modified Eagle Medium, fetal calf serum (FCS), penicillin/streptomycin (P/S) mixture, and L-glutamine were also obtained from PAN Biotech GmbH. The commercially available heparins were purchased from the following sources: unfractionated heparin-sodium (UFH, 1): Heparin-5000-ratiopharm from Ratiopharm GmbH, Ulm, Germany; LMWHs: tinzaparin (2), Innohep^®^ from LEO Pharma, Ballerup, Denmark, dalteparin (3), Fragmin^®^ from Eurim Pharma GmbH, Saaldorf-Surheim, Germany, nadroparin (4), Fraxiparin^®^, Berenga Arzneimittel, Baden-Baden, Germany; enoxaparin (5), Clexane^®^ from Sanofi-Aventis Deutschland GmbH, Frankfurt, Germany; fondaparinux (6), Arixtra^®^ from Aspen Pharma Trading Ltd, Dublin, Ireland. The non-commercial compounds 7-15 were synthetized as described (see **Table 1** and **Figure 2** for details) (19).

**Table 1:** Investigated heparins and their inhibitory potency at human NPP1.

No.	Compound	(Mean) MW (KDa)	SuD^a^	Structural Information	IC_50_ ± SD (µM)	IC_50_ ± SD (µg/ml) [or (IU/ml)]
**1**	Heparin sodium (UFH)	8-25	2.7	25-40 m.u.^b^	ca. 0.503 ± 0.100^c^	8.29 ± 1.74 µg/mL (4.15 ± 0.87 IU/mL)
**2**	Tinzaparin	6.5	2.5	~ 20 m.u.	ca. 2.80 ± 0.60^c^	18.4 ± 4.2^d^ (9.21 ± 2.10 IU/mL)
**3**	Dalteparin	6	2.6	~ 17 m.u.	24.7 ± 7.2	148 ± 52
**4**	Nadroparin	4.5	2.5	14-16 m.u.	42.7 ± 12.7	192 ± 57
**5**	Enoxaparin	4.5	2.3		8.86 ± 1.40	52.1 ± 6.3
**6**	Fondaparinux	1.7	8	pentasaccharide	2.94 ± 0.61	4.99 ± 0.98
**7**	G9694	16.6	2.2	RO heparin (reduced oxyheparin); repetitive disaccharide unit (600 Da)	15.4 ± 2.13	256 ± 36
**8**	G5945	16.6	1.1	2-O-desulfo-heparin (desulfated); repetitive disaccharide unit (530 Da)	29.7 ± 0.005^d^	487 ± 0^d^
**9**	G6658	15.4	0.7	6-O-desulfo-heparin (85% 6-OH; 52% non-sulfated uronic acid); repetitive disaccharide unit (490 Da)	0.79 ± 0.14	12.1 ± 2.1
**10**	A3875-G6	3.6	2.6	dodecasaccharide fraction of dalteparin (dalte)	3.31 ± 2.30	11.9 ± 8.1
**11**	A3875-H6	3	2.6	dalte-decasaccharide	16.7 ± 4.8	50.0 ± 14.3
**12**	A3875-I6	2.4	2.7	dalte-octasaccharide	4.48 ± 0.89	10.7 ± 2.1
**13**	G4271A	3	nd^e^	depolymerization product of RO-heparin and 2-O-desulfo-heparin	19.7 ± 3.2	59.1 ± 9.5
**14**	G4271B	≤ 3	nd	depolymerization product of RO-heparin and 2-O-desulfo-heparin	73.5 ± 22.9	220 ± 69
**15**	G4185	3	nd	Mixture of G4271A + G4271B	3.46 ± 0.03	10.4 ± 0.1

a SuD, sulfation degree (average number of sulfate groups per disaccharide repeating unit; for fondaparinux the total number of sulfate groups is given).

b m.u., monosaccharide unit.

c Estimated; 1 IU equals to 0.002 mg of heparin; a mean value of 16.5 kDa for heparin-sodium was considered for calculation.

d IC_50_ value extrapolated.

e nd, not determined.

### 2.2 NPP1 assay

Concentration-inhibition curves of the test compounds at human NPP1 were performed with ATP as substrate at a final concentration of 400 μM. Several different dilutions of the test compounds were prepared in assay buffer (10 mM *N*-cyclohexyl-2-aminoethanesulfonic acid (CHES), 2 mM CaCl_2_, 1 mM MgCl_2_, pH 9.00) and incubated with 20 ng of human recombinant soluble NPP1 (Val191 - Leu591) expressed in murine myeloma NS0 cells obtained from R&D Systems GmbH (Wiesbaden, Germany, purity 95%, purified by using an *N*-terminal His-tag). For initial screening and selectivity analysis, NPP1 prepared as previously described (20), diluted in assay buffer, was used. The mixture of enzyme, substrate and test compound or buffer without test compound was incubated for 30 min at 37°C, and the reaction was terminated by heating at 90°C for 5 min. Capillary electrophoresis (CE) analysis was carried out to analyze the formation of the product. Data collection and peak area analysis were performed by the P/ACE MDQ software 32 KARAT obtained from Beckman Coulter (Fullerton, CA, USA). A polyacrylamide-coated capillary was used [30 cm (20 cm effective length) × 50 μm (id) × 360 μm (od) purchased from Chromatographie Service GmbH (Langerwehe, Germany)]. Samples were injected electro-kinetically by applying a voltage of -6 kV for 30 s. Finally, analytes were separated by applying a separation voltage of -20 kV, and detected by UV at 260 nm. The *IC_50_* values were determined by nonlinear curve fitting using the GraphPad Prism Software 7.0. The mechanism of inhibition at human NPP1 was determined employing different concentrations of the investigated compounds 6 (at 0, 0.4, 4.0 µM), and 7 (at 0, 0.4 and 40 µM) versus four different substrate concentrations ranging from 50 to 500 μM of ATP. The assay procedure and operation conditions were the same as described above. The experiment was conducted twice, each in triplicates. A Lineweaver-Burk was calculated using GraphPad Prism 7.0 for predicting the inhibition type of the compound.

### 2.3 NPP3 and NPP5 assay

Compounds 1, 2, 5, 6, and 7 were screened at different concentrations using 400 μM of *p*-nitrophenyl thymidine monophosphate (*p*-Nph-5´-TMP) as substrate to study potential inhibition of NPP3 and NPP5 using a colorimetric assay as previously described (21). Soluble and purified human NPP3, prepared as previously described (22), and human NPP5 (23) were diluted in the assay buffer (50 mM TRIS HCl, 2 mM CaCl_2_, and 0.2 mM ZnCl_2_, pH 9.00), and 0.09 µg of NPP3, or 0.4 µg of NPP5 were employed. The mixtures were incubated for 30 min at 37°C in case of NPP3, and for 100 min at 37°C in case of NPP5, and the reactions were subsequently terminated by adding 20 μL of 1.0 N aq. NaOH solution. The assay is based on the enzymatic ester hydrolysis of *p*-Nph-5’-TMP which results in the formation of the yellow *p*-nitrophenolate anion. The absorption was measured at 400 nm using a BMG PheraStar FS plate reader (BMG Labtech GmbH, Ortenberg, Germany). Each analysis was repeated three times with triplicate measurements.

### 2.4 NPP4 assay

Selectivity studies on NPP4 were performed using a published luminescence-based assay versus 20 μM of diadenosine tetraphosphate (Ap_4_A) as a substrate and human soluble NPP4 (24). The assay buffer consisted of 10 mM HEPES, 1 mM MgCl_2_, and 2 mM CaCl_2_ (pH 8.00). The enzyme reaction was started by adding 0.14 μg of purified human NPP4 to the reaction mixture, which was incubated at 37°C for 90 min. The released product ATP was quantified using the luciferin-luciferase reaction. The firefly luciferase reacts with D-luciferin in the presence of the formed ATP and Mg^2+^. The resulting luminescence is a measure of the enzymatic activity, and the luminescence was read at 560 nm using a microplate reader (BMG PheraStar). Three independent experiments were performed, each in duplicate measurements; positive and negative controls were included.

### 2.5 NTPDase assays

For monitoring the activity of human NTPDases (subtypes 1, 2, 3 and 8) in the presence of test compounds, a CE-based assay system was used, according to a published procedure (11, 25). The following concentrations of test compounds were investigated in initial screening studies: 63 IU/mL of compounds 1 and 2, 30 µM of compound 6. For selectivity studies, the following concentration were employed: 50 IU/mL of 1, 12 µM of 6, and 48 µM of 7. The amount of enzyme was selected according to previously performed enzymatic titration, and adjusted to ensure a 10–20% conversion rate. Enzymes were obtained by transfection of COS-7 cells using plasmids encoding human NTPDase1 (GenBank accession number U87967) (26), NTPDase2 (NM_203468) (27), a kind gift of Dr. A.F. Knowles (San Diego, CA), NTPDase3 (AF034840) (28), a kind gift of Dr. T.L. Kirley (Cincinnati, OH) and human NTPDase8 (AY430414) (29) and purified as previously described (30, 31). The reaction buffer consisted of 10 mM HEPES, 2 mM CaCl_2_, and 1 mM MgCl_2_, pH 7.4. In the CE assay, the selected substrate concentration (ATP) was 100 µM for all studied human NTPDases (-1, -2, -3 and -8). Mixtures of enzymes with substrate and test compounds were incubated at 37°C for 30 min, and the enzymatic reaction was stopped by heating for 10 min at 95°C. The released products were separated by CE and quantified by their UV-absorption at 260 nm with ADP and AMP as external standards, as previously described (11); positive and negative controls were included.

### 2.6 CD73 assay

The assay was performed essentially as previously described (32). The enzymatic reaction was performed by mixing 0.36 ng of human CD73 (33) with test compound in assay buffer (25 mM Tris, 140 mM sodium chloride, 25 mM sodium dihydrogen phosphate, pH 7.4) and 5 µM of [2,8-^3^H]AMP [(specific activity 7.4x10^8^ Bq/mmol (20 mCi/mmol)), American Radio-labeled Chemicals, MO, USA, distributed by Hartmann Analytic, Germany]. Compound 7 was used at 800 µg/mL and compound 6 at 20 µg/mL for the selectivity test, while for initial screening compound 5 was tested at a concentration of 55 µM and 6 at 30 µM, and 1 and 2 were tested at 50 IU/mL (**Supplementary Figure 1**). The enzymatic reaction was performed for 25 min at 37°C in a shaking water bath. Then, 500 µL of cold precipitation buffer (100 mM lanthanum chloride, 100 mM sodium acetate, pH 4.00) was added to stop the reaction and to facilitate precipitation of free phosphate and unconverted [2,8-^3^H]-AMP. After the precipitation was completed (after at least 30 min on ice), the mixture was separated by filtration through GF/B glass fiber filters using a cell harvester (M-48, Brandel, MD, USA). After washing each reaction vial three times with 400 µL of cold (4°C) demineralized water, 5 mL of the scintillation cocktail (ULTIMA Gold XR, PerkinElmer, MA, USA) was added and the radioactivity was measured by scintillation counting (TRICARB 2900 TR, Packard/PerkinElmer; counting efficacy: 49-52%). Positive and negative controls were included.

### 2.7 mRNA gene expression of ectonucleotidases in human glioblastoma cell lines

The mRNA isolated from several primary human glioblastoma cell specimens, namely 46Z, 78Z, 106Z, 138Z, from adults’ human brain-derived neural progenitors, 155Z and 167Z, and mRNA isolated from human glioblastoma cell lines T98G, LN229, U138, and U87, were studied with respect to gene expression of ectonucleotidases. A two-step RT-qPCR (quantitative reverse transcription polymerase chain reaction) technique was used. The reverse transcription and PCR amplification steps were performed in two separate reactions. mRNA samples from the cell lines (1 µg) were treated with 1 µL of DNase I (NEB; Cat. No. M0303S) for the removal of contaminating genomic DNA, incubated at room temperature, followed by addition of 25 mM of EDTA (ethylenediaminetetraacetic acid) and incubated at 65°C for 10 min. Then, the reverse transcription was achieved with the iScript cDNA Synthesis Kit (Bio-Rad; Cat. Num: 170-8891) using 0.5 µg of reaction mix available from the manufacturer, 1 µL of reverse transcriptase and RNA templates. Subsequently, 1 µL (5000 U/µL) of RNase H digest (NEB; Cat. No. M0297S) was added to each cDNA sample and incubated for 20 min at 37°C. Finally, the cDNA samples were diluted to a final concentration of 2.5 ng/µL, and qPCR was performed using SYBR Green (Bio-Rad iQ SYBR Green Supermix; Cat. No. 170-8887); the cDNA (5 ng) was mixed with 250 nM of the respective sequence-specific primers of the different investigated ectonucleotidases, which have been checked for their efficiency (**Supplementary Figure 3**), and positive and negative controls together with reference genes were tested in parallel. The qPCR cycle started with polymerase activation and DNA denaturation at 95°C for 3 min, followed by 49 cycles of amplification at 95°C for 20 s, 60°C for 20 s and 40 s at 68°C, finishing with 95°C for 30 s and 30 s at 65°C; melt curve: 60°C to 95°C, increments of 0.5°C and finally the plate was read (hard shell, thin wall white 96-well plates from Bio-Rad Cat. No. HSP9655). The data were collected and analyzed using CFX manager 2.0 software provided by Bio-Rad using their system C1000Thermal Cycler + CFX96 Real-Time System.

### 2.8 cDNA sequencing of NPP1 in U87 cells

Total RNA was extracted from 5 × 10^6^ U87 cells using the Direct-zol RNA Miniprep Kit (Zymo Research; Cat. No. R2050). The isolated RNA was subsequently used for first strand cDNA synthesis, which was performed with the SuperScript Reverse Transcriptase IV kit (ThermoFisher Scientific; Cat. No. 18091050). The reverse transcription was carried out according to the manufacturer’s instruction with 1 µg of total RNA and 50 pmol of oligo d(T)20 primer. PCR amplification of the NPP1 cDNA was achieved with the gene-specific forward primer 5’-GCCAAGGACCCCAACACCTATAAAG-3’ that binds downstream of the initial GC-rich region of the coding sequence (CDS) of NPP1 thereby omitting the first 204 bp of the CDS, and the following reverse primer 5’-CGACAGTTTCTGAACAATGCAATAG -3’. The PCR product was purified and extracted from a 1% agarose gel using the Zymoclean Gel DNA Recovery Kit (Zymo Research; Cat. No. D4001). Afterwards, the purified cDNA was cloned into the pJet1.2 vector (ThermoFisher Scientific CloneJET PCR Cloning Kit; Cat. No. K1231) following the manufacturer’s blunt-end cloning protocol. Then, the reaction product was transformed into competent DH5α cells. Multiple clones were sequenced using the standard pJet1.2for and pJet1.2rev primers, as well as the NPP1 gene specific primer 5’-GGATTGTATCCAGAATCTCATGGC-3’. The sequences were analyzed using SnapGene software (www.snapgene.com) and Clustal Omega (34). For sequencing results see **Supplementary Figure 4**.

### 2.9 Membrane preparation of U87 cells

U87 glioblastoma cells were grown in cell culture dishes until reaching a fully covered confluent cell layer. Cells were cultivated at 37°C and 5% CO_2_ in DMEM with additions of 10% FCS, 1% penicillin/streptomycin (P/S) mixture and 1% L-glutamine. When confluent, the cell culture media was carefully removed and cells were frozen at -20°C overnight. The cells were then scraped off with a cell scraper using 2-3 mL of buffer (5 mM Tris, 2 mM EDTA, pH 7.4), homogenized and centrifuged for 10 min at 1000 g. The supernatant was collected and centrifuged for 60 min at 48000 g and 4°C. The pellet was then resuspended twice with Tris buffer, pH 7.4, followed by vortexing and centrifugation. The whole procedure was performed on ice. Finally, the pellet was resuspended and homogenized. A protein concentration of 1.5 mg/mL was determined with the Bradford method (35), and the protein was kept at -20°C until used. The enzymatic assay was performed by incubating the membrane preparation and the substrate at 37°C and 500 rpm for 30 min, followed by heat inactivation at 95°C for 5 min. For the determination of kinetic parameters, several substrate concentrations (from 6 to 500 µM of ATP or AMP) were utilized and incubated with 10 µg of membrane preparation for 30 min at 37°C, followed by heat inactivation. Capillary electrophoresis (CE) was used for analysis and quantification of the enzymatic product according to published procedures (11, 36). For the determination of the enzymatic activity 400 µM of ATP were employed, and three different amounts of membrane preparation (6, 10 and 30 µg of protein) were utilized. A positive control using the known NPP1 inhibitor PSB-POM-141 (20 µM) was performed (19). Data were obtained from three independent experiments each in duplicate measurements. Subsequently, compounds 6 and 7 were studied for their potency to reduce adenosine formation, at 3 and 30 µM (Compound 6, fondaparinux), and at 35 µM (Compound 7, G9694). Controls were run in parallel.

### 2.10 U87 cell culture, and cell-based assay

U87 human glioblastoma cells were grown in cell culture flasks until reaching a fully covered confluent cell layer. Cells were cultivated at 37°C and 5% CO_2_ in DMEM with additions of 10% FCS, 1% penicillin/streptomycin and 1% L-glutamine. The cells were rinsed with phosphate-buffered saline (PBS) and detached with EDTA. Thereafter, they were counted by a Cell Counter CASY^®^ 1 Model TT (Schärfe System GmbH, Reutlingen, Germany) to retrieve the number of viable cells, and washed three times with PBS before they were resuspended in Krebs-HEPES buffer at a concentration of 10^6^ cells/mL (37). Then, 1 mL of cell suspension was transferred into a 24-well plate. The cells were pretreated with the nucleoside transport inhibitor dipyridamole (20 µM) for 30 min (38). Adenosine deaminase (ADA) activity was inhibited by the addition of 1 µM of the inhibitor EHNA (erythro-9-amino-β-hexyl-α-methyl-9*H*-purine-9-ethanol hydrochloride) (39). Heparins, their non-anticoagulative analogs, and the known ectonucleotidase inhibitor PSB-POM-141 (20) were added. The samples were incubated with 300 µM of ATP for 3 h at 37°C and 5% CO_2_. Then, an aliquot of 300 µL of solution from each vial was transferred into 1.5 mL Eppendorf tubes and heated at 95°C for 10 min to inactivate enzymes to avoid further nucleotide degradation. Before performing capillary electrophoresis (CE) measurements, the samples were centrifuged at 600 g to remove insoluble material like cell debris from the supernatant. Until measurement, samples were stored at -20°C. Nucleotides (ATP, ADP, AMP, cAMP) and nucleosides (adenosine, inosine, uridine), which were used as standard compounds to monitor efficient separation by CE, were dissolved in Krebs-HEPES buffer at a concentration of 10 µM and stored at -20°C (37). All CE measurements were performed on a P/ACE capillary electrophoresis system MDQ glycoprotein (Beckman Coulter Instruments, Fullerton, CA, USA) using a fused silica capillary without coating, according to a published method (40). The capillary dimensions were 40 cm in length (30 cm effective length to detection window) and 75 µm in diameter. Preliminary to the measurement, the capillary was rinsed with 0.1 N aq. NaOH solution, Millipore water and running buffer to establish consistent conditions. The running buffer consisted of sodium dodecyl sulfate (SDS, 100 mM) and disodium hydrogen phosphate (10 mM) at pH 8.0. SDS creates a pseudo-stationary phase, which enables the separation of the uncharged nucleosides. The hydrodynamic injection for 30 s with a pressure of 0.1 psi. A constant current of -90 µA, 10 kV and a pressure of 0.1 psi were required for the separation. The detection was performed by a UV diode array detector which allowed to identify the UV spectra of the nucleosides and nucleotides at a wavelength of λ = 260 nm (40).

### 2.11 Induced Treg differentiation assay

Human CD4^+^ T cells were extracted from buffy coats by positive selection using the StraightFrom™ Buffy Coat CD4 MicroBead Kit (Miltenyi Biotec, Bergisch Gladbach, Germany). CD4^+^ T cells were stimulated by ImmunoCult™ Human CD3/CD28/CD2 T Cell Activator at day 1. At days 1 and 3, the indicated compounds or U87 cell supernatant, either of untreated or of heparin-treated cells were added to CD4^+^ T cells. Cells were harvested at day 5 and were investigated for CD25 and FoxP3 expression. Therefore, cells were washed once with staining buffer (1% BSA, 0.01% NaN_3_ in PBS). To avoid nonspecific binding, cells were blocked for 15 min with 3% BSA in PBS. Anti-CD25 antibody (PerCP/Cyanine5.5 anti-human CD25 antibody, RRID : AB_2125478, BioLegend Inc, San Diego, CA, USA) was added and the cells were incubated for 30 min in the dark. Subsequently, cells were washed once with staining buffer and fixed in 150 µL of fixation buffer (3% paraformaldehyde (PFA), 0.1% saponin, and 0.5% Tween-20 in Dulbecco's phosphate-buffered saline) for 30 min at 4°C. Samples were washed once with permeabilization buffer (0.5% saponin, 0.5% Tween-20 in staining buffer). Next, cells were permeabilized in 75 µL of permeabilization buffer for 30 min at 4°C. Samples were spiked with anti-FoxP3 antibody, fluorescein isothiocyanate ((FITC-labeled), RRID : AB_439752, BioLegend Inc.) diluted in 25 µL of permeabilization buffer. After an incubation period of 30 min at 4°C, cells were washed twice with 150 µL permeabilization buffer and finally resuspended in 500 µL of staining buffer and transferred into a suitable plate. Samples were measured in triplicates using the Guava^®^ easyCyte HT Flow Cytometer (Merck Millipore, Billerica, MA, USA). Compensation and FMO (fluorescence minus one) controls were conducted. The gating strategy is displayed in **Supplementary Figure 6**. Data were evaluated using the FlowJo™ v10.5.3 Software (RRID : SCR_008520, BD Life Sciences, Franklin Lakes, NJ, USA) and Graphpad PRISM 8.4.0.

## 3 Results

Heparin is a complex mixture of naturally occurring anionic, sulfated glycosaminoglycans extracted from pig gut mucosa. Unfractionated heparin (UFH, 1, heparin sodium) whose molecular weight is ranging from 8-25 kDa, is a linear polymer made up of 1→4 linked disaccharide repeating units, consisting of α-D-glucosamine, 2-deoxy-2-sulfamino-D-glucose, 2-acetamido-2-deoxy-D-glucose, α-L-iduronic or β-D-glucuronic acid with 2.7 (average number) sulfated groups per disaccharide repeating unit. Low molecular weight heparins (LMWHs) are obtained from UFH by chemical or enzymatic depolymerization resulting in polysaccharide fragments of smaller size. Three commercially available heparins, UFH (1), the LMWHs tinzaparin (2), and enoxaparin (5), and the synthetic pentasaccharide fondaparinux (6) (for structures see **Figure 2**) were initially evaluated as potential inhibitors of prominent members of the ectonucleotidase family, namely NTPDase1 (CD39) and its isoenzymes NTPDase2, -3, and -8, NPP1 and its isoenzyme NPP3, and ecto-5’-nucleotidase (CD73). We observed almost complete inhibition of human NPP1 by all four compounds, at a concentration of 63 IU/mL for heparin-Na (1) and tinzaparin (2), and at 250 µg/mL and 50 µg/mL of enoxaparin (5) and fondaparinux (6), respectively. For the other investigated ectonucleotidases, only low or very low inhibition was observed under the same conditions, indicating selectivity of the heparins for NPP1 (**Supplementary Figure 1**).

### 3.1 NPP1-inhibitory potency

**Figure 3:**
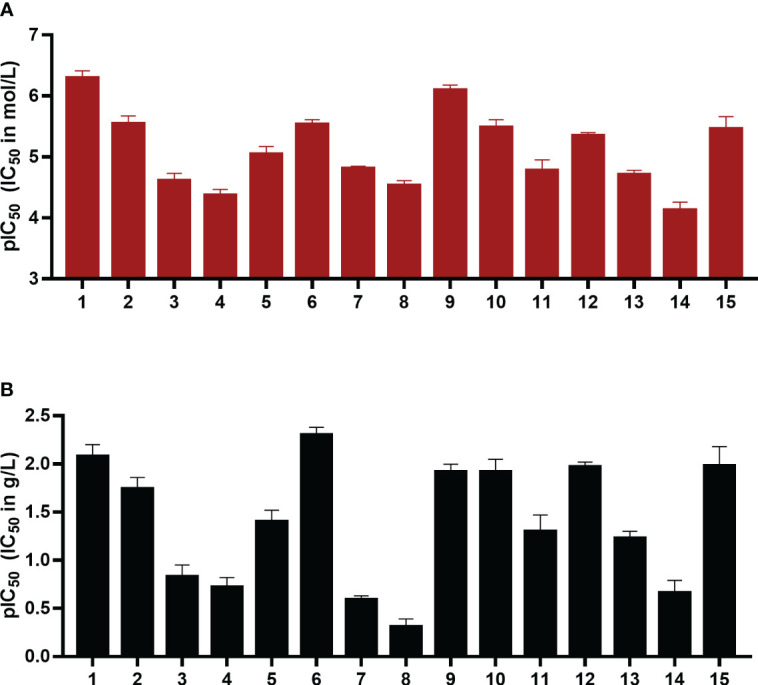
pIC_50_ values in mol/L **(A)** and g/L **(B)** of compounds 1-15 as inhibitors of the human ectonucleotidase NPP1 are shown. Error bars represent SD values. For details see **Table 1**. ATP was used as a substrate, and a CE-UV-assay was used for detection and quantification of the enzymatic reaction product AMP. For details see Experimental Section.

Due to the observed selective inhibition of NPP1 by the initially tested heparins, we decided to study a broader range of heparin derivatives as potential inhibitors of NPP1. In addition to UFH (heparin sodium salt; compound 1) and tinzaparin (compound 2), dalteparin (sodium salt; compound 3), and nadroparin (calcium salt; compound 4) were applied as further representatives of LMWHs, in addition to enoxaparin (compound 5), displaying decreasing molecular weights in this order. Fondaparinux (compound 6) is a fully synthetic anticoagulant, which is based on the pentasaccharide sequence that represents the antithrombin binding site of heparin that is responsible for the anticoagulant effect. In addition to commercially available drugs, we tested the non-commercial heparin derivatives 7-15 (see **Figure 2**). To study the impact of the molecular size on NPP1 inhibitory potency, we applied defined fractions of dalteparin (compound 10-12) representing an octa-, deca- and dodecasaccharide fraction of this LMWH. Furthermore, chemically modified UFH derivatives were included that were synthesized to reduce or abolish the anticoagulant activity. Thus, we applied compound G9694 (7), a glycol-split derivative with ring-opening of non-sulfated uronic acids (reduced oxyheparin), G5945 (8) as a 2-O-desulfated heparin, and compound 9 that is desulfated in position 6 of glucosamine. Compounds 7, 8 and 9 have similar molecular weights of around 16 kDa, with degrees of sulfation of 2.2, 1.1, and 0.7, respectively. Furthermore, the 2-O-desulfated heparin has been size-fractionated yielding compounds G4271A (13), G4271B (14), and G4185 (15), which is a mixture of 13 and 14. Altogether, 15 heparin derivatives were pharmacologically characterized, and their potencies in inhibiting ATP hydrolysis by human NPP1 were determined using a capillary electrophoresis(CE)-based assay (see **Table 1**, **Figure 3**, and **Supplementary Figure 2**).

The IC_50_ values ranged from 0.503 µM for UFH (compound 1) to 73 µM for G4271B (compound 11). Among the clinically used heparins, UFH (compound 1) and fondaparinux (6) appeared to be the most potent ones in inhibiting NPP1 activity with IC_50_ values of 8.29 µg/mL and 4.99 µg/mL, respectively, followed by tinzaparin (2) with an IC_50_ value of 18.4 µg/mL. These compounds have 2.7, 3.2 and 2.5 sulfate groups per disaccharide repeating unit on average. LMWHs possess a lower total number of sulfate groups compared to UFH; their IC_50_ values ranged from 20 to 190 µg/mL. Among the LMWHs, dalteparin (3) exhibits a MW of 6 kDa while nadroparin (4) and enoxaparin (5) have a lower MW of 4.5 kDa; compound 5 was found to be almost 5 times more potent than compounds 3 and 4.

Desulfation of position 6 (G6658, 9) instead of position 2 (G5945, 8) was advantageous, compound 9 being 35-fold more potent than 8. Compounds 10, 11, and 12, which differ by two or four units in length, displayed IC_50_ values in a similar range, however a dodecasaccharide of 3.6 kDa (compound 10) showed stronger inhibition of NPP1 activity compared with 8 or 10 units (G9694, 7). The non-anticoagulant heparin derivative (reduced oxyheparin) exhibited an IC_50_ value of 30 µM, thus, the ring-opening modification did not interfere with the compound’s inhibitory activity at NPP1. For direct comparison, the *p*IC_50_ values are depicted in **Figure 3**. Due to the large differences in the molecular weights of the compounds, a comparison based on g/L (**Figure 3B**) appears to be more meaningful than a comparison of molar concentrations (**Figure 3A**). These data confirm that different heparin derivatives are suitable to block NPP1 activity, and the size of the oligo/polysaccharides does not seem to be the main determining factor for inhibitory activity. The potency of the heparin derivatives depends significantly on charge density and is unrelated to anticoagulant activity. This offers the opportunity to develop heparin derivatives and analogs with improved NPP1 inhibition but lacking anti-coagulant activity.

### 3.2 Selectivity versus other ectonucleotidases

**Figure 4:**
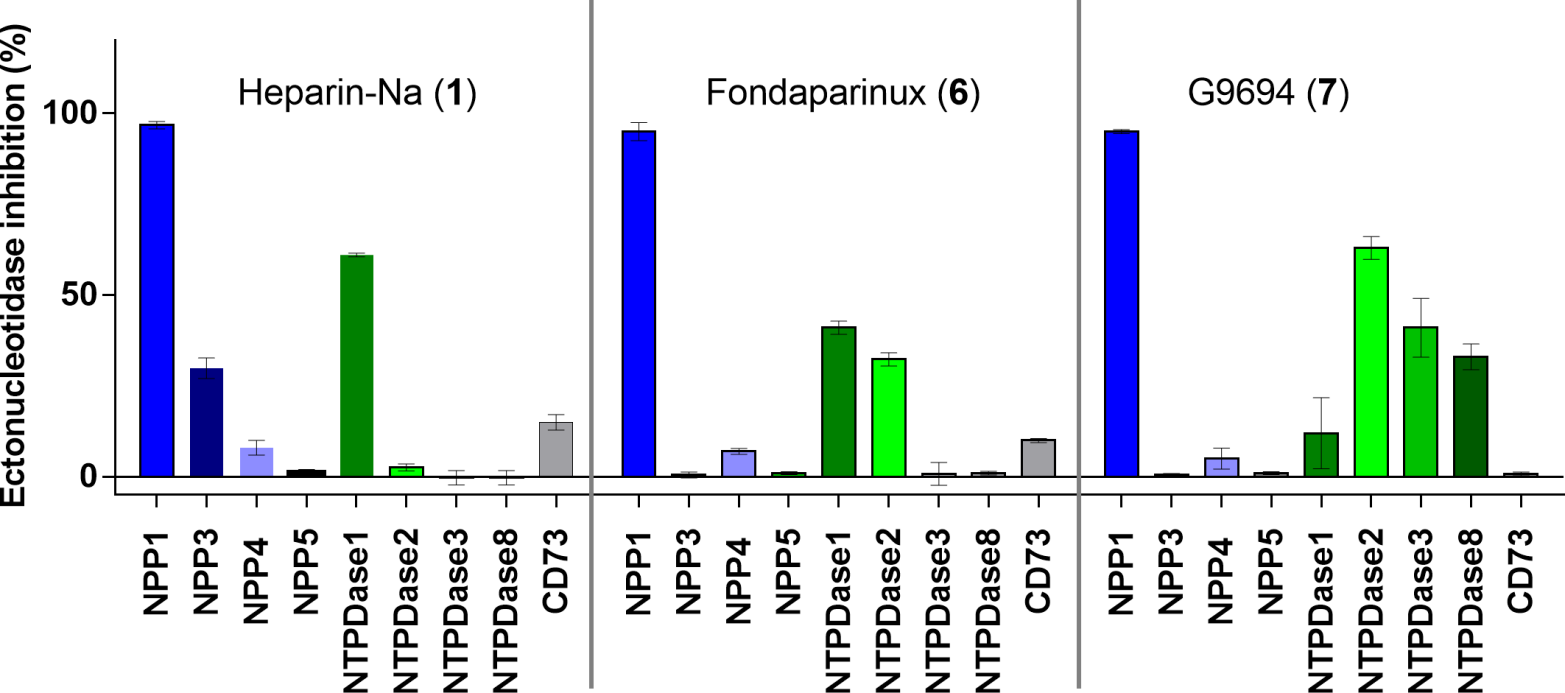
Ectonucleotidase inhibition by UFH (1), fondaparinux (6) and G9694 (7) selected as representatives of structurally diverse heparins. A concentration was selected by which full inhibition at NPP1 was observed. Compound 1 was tested at 50 IU (roughly 6 µM), compound 6 at 20 µg/ml (12 µM) and compound 7 at a concentration of 800 µg/mL (48 µM). NPP1 and NTPDase1, 2, 3 and 8 activities were analyzed using a CE-based assay employing ATP as a substrate; NPP3 and NPP5 were investigated using *p*-Nph-5′-TMP as a substrate; NPP4 was tested versus Ap_4_A as a substrate using a luciferase assay; for CD73 activity a radioactive assay was performed. The data are normalized with respect to positive (100%) and negative (0%) controls of each enzyme, for details see Experimental Section.

Next, we studied the selectivity of some heparins versus other members of the ectonucleotidase family. UFH (1), fondaparinux (6), and G9694 (7) were selected representatives for anticoagulant and non-anticoagulant heparin derivatives (**Figure 4**). A concentration was selected by which full inhibition of NPP1 was observed. All three compounds showed high selectivity for NPP1 versus other NPP subtypes, NPP3, NPP4, and NPP5, and versus the ecto-NTPDases NTPDases1, NTPDase2, NPTDase3, and NTPDase8, as well as the ecto-5’-nucleotidase CD73. Compound 7 (G9694), tested at 800 µg/mL (48 µM), inhibited NTPDase1 (CD39) by 60%, and slightly inhibited NTPDase3 and -8 by about 35%, but no effect was observed on the other enzymes. Fondaparinux (6), tested at 20 µg/mL (12 µM), showed ~35% inhibition of NTPDase1 and -2, but no effect on the other investigated enzymes was observed, while UFH (1), tested at 50 IU/mL (roughly 6 µM), inhibited only NTPDase1, blocking its activity by about 60% - in addition to full NPP1 inhibition.

### 3.3 Determination of inhibition type

**Figure 5:**
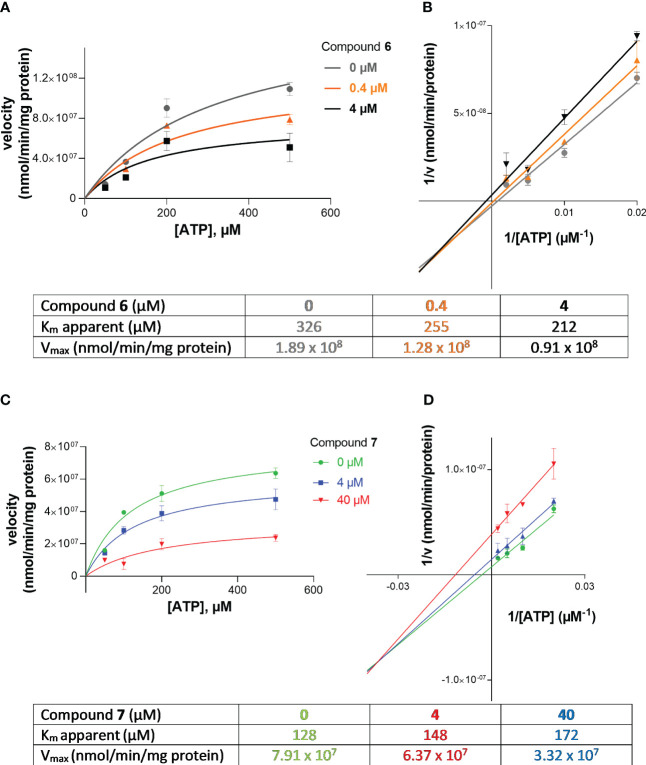
Investigation of the inhibition type of inhibitors 6 (fondaparinux) and 7 (G9694) at human NPP1. In **(A, B)**, the Michaelis-Menten curves are shown without and in the presence of different concentrations of the inhibitor. Lineweaver-Burk plots are shown in **(C, D)** indicating the inhibition type. The kinetic parameters of ATP hydrolysis by NPP1, in the absence and presence of inhibitors 6 and 7, are provided in the table. The results indicate a mixed type of inhibition. Data were obtained from three independent experiments, each performed in duplicate.

Next, we investigated the mechanism of inhibition for compound 7 (G9694) and compound 6 (fondaparinux) as group representatives for semisynthetic non-anticoagulative heparin derivatives and for commercially available anticoagulants. ATP hydrolysis by NPP1 was determined in the absence and in the presence of the inhibitors. Four different concentrations of the substrate ATP were employed (50, 100, 200 and 500 µM), each tested in the presence of 0, 0.4, and 4 µM of fondaparinux (6), or 0, 4, and 40 µM of G9694 (7). For the determination of the inhibition type, Lineweaver-Burk plots were computed (**Figures 5C, D**), and the kinetic parameters of ATP hydrolysis by NPP1, in the absence and presence of inhibitors 6 and 7, were calculated (**Figures 5A, B**). The results indicated a mixed-type of NPP1 inhibition by these heparin derivatives (**Figure 5**). This means that the investigated compounds bind to an allosteric site (a site different from the active site where the substrate binds), and the inhibitors may bind to the enzyme without substrate or to the substrate-bound enzyme. The presence of a mixed-type inhibitor decreases the apparent maximum enzyme reaction rate (see V_max_ values in **Figure 5**). Furthermore, a mixed-type inhibitor can either lead to decreased K_m_ values (see compound 6) indicating preferred binding to the enzyme-substrate complex, or to increased K_m_ values indicating preferred binding to the free enzyme (see inhibitor 7).

### 3.4 Identification of cancer cell lines with high NPP1 expression

To test the compounds in a more complex system, we searched for a native cell line with high NPP1 expression. Thus, mRNA expression analysis of ectonucleotidases in several tumor cell lines was carried out. Altogether, 10 different cell lines were investigated with respect to 8 genes encoding members of the ectonucleotidase family. Expression of NPP1 was previously reported to be increased in human astrocytic brain tumors (glioblastoma), and NPP1 expression was observed to increase with tumor grade (40). NPP1 was also shown to be expressed in N2a mouse neuroblastoma cells, the expression level significantly decreasing upon differentiation into a neuronal-like phenotype (41). Therefore, we studied the gene expression of adult human brain-derived neural progenitor cells (155Z and 167Z), primary human glioblastoma cell specimens (106Z, 138Z, 46Z, 78Z) and established human glioblastoma cell lines (LN229, T98G, U138 and U87). mRNA expression for NPPs (*ENPP-1*, *-2* and *-3*), NTPDases (*ENTPD1*, *-2*, *-3* and *-8*) and 5’-ectonucleotidase/CD73 (*NT5E*) was determined by RT-qPCR (quantitative reverse transcription polymerase chain reaction).

In the initially performed broader screening (**Table 2A**) we observed very low or no mRNA expression for NTPDases, while relatively high mRNA expression for NPP1 was detected in all investigated cell lines, along with very high mRNA expression for ecto-5’-nucleotidase (CD73). Subsequently, the number of cell lines was reduced, and we specifically studied the expression of ENPP1, ENTPD1, -2 and -3, and NT5E mRNA. Indeed, we confirmed that 167Z, LN229, U87, 138Z and 46Z cells all have high mRNA expression levels encoding for NPP1 and CD73 compared to very low or no mRNA expression encoding for NTPDases. Based on the observed expression of the genes of interest in these cell lines, we selected the glioblastoma cell line U87 for further experiments. U87 is an established human glioblastoma cell line that expresses NPP1 and CD73, with no significant expression of any of the other investigated ectonucleotidases, thus making it suitable for our purpose (see **Table 2B**).

Table 2APreliminary quantitative mRNA expression analysis of ectonucleotidases in human cell lines determined by qPCR.^a^

**Table:** 

NPPs				NTPDases				CD73
Cell line	Cq ENPP1	Cq ENPP2	Cq ENPP3	Cq ENTPD1	Cq ENTPD2	Cq ENTPD3	Cq ENTPD 8	Cq NT5E
155Z	26.68	26.81	≥35	32.93	≥35	≥35	≥35	21.71
167Z	26.15	26.09	≥35	31.38	≥35	≥35	≥35	22.53
LN229	29.62	25.99	≥35	≥35	≥35	≥35	≥35	27.00
T98G	33.82	31.58	≥35	≥35	≥35	≥35	≥35	31.06
U138	27.95	34.28	≥35	≥35	≥35	≥35	≥35	28.65
U87	27.07	25.53	≥35	≥35	≥35	≥35	≥35	23.76
106Z	28.68	36.28	≥35	33.00	31.93	30.01	≥35	28.06
138Z	27.78	28.53	≥35	≥35	≥35	≥35	≥35	25.28
46Z	27.45	29.15	≥35	≥35	30.94	31.60	≥35	25.04
78Z	27.93	30.10	≥35	≥35	≥35	≥35	≥35	25.54

**Table:** 

**Cell lines:**
**adult human brain-derived neural progenitor cells**
**human glioblastoma cell line**
**primary human glioblastoma cells**

**Table:** 

**Cq >35**	**no mRNA expression**
**Cq = 30-34**	**very low mRNA expression**
**25<Cq ≤ 30**	**low mRNA expression**
**Cq ≤ 25**	**high mRNA expression**

a Data from initial screening (Cq, n=4) where the genes of interest were investigated in 10 different cell lines. Cq, quantitation cycle; ENPPs, ectonucleotide pyrophosphatase/phosphodiesterases; ENTPDases, ectonucleoside triphosphate diphosphohydrolases; NT5E, ecto-5’-nucleotidase (CD73); GAPDH, glyceraldehyde-3-phosphate dehydrogenase.

Table 2BQuantitative mRNA expression analysis of selected ectonucleotidases in specific human cell lines determined by qPCR.^a^

**Table:** 

	NPPs	NTPDases			CD73	Reference genes	
Cell line	Cq ENPP1	Cq ENTPD1	Cq ENTPD2	Cq ENTPD3	Cq NT5E	Cq ß-actin	Cq GAPDH
167Z	26.42 ± 0.27	30.68 ± 0.70	≥35	≥35	22.45 ± 0.09	18.19 ± 0.48	19.38 ± 0.10
LN229	29.51 ± 0.12	≥35	≥35	≥35	25.86 ± 1.15	19.71 ± 1.01	19.93 ± 0.44
U87	26.67 ± 0.40	≥35	≥35	≥35	23.06 ± 0.70	18.52 ± 0.81	19.92 ± 0.38
138Z	27.59 ± 0.19	≥35	≥35	≥35	25.19 ± 0.09	19.03 ± 0.15	19.50 ± 0.13
46Z	28.05 ± 0.60	≥35	31.15 ± 0.21	32.00 ± 0.39	25.00 ± 0.05	19.13 ± 0.23	19.66 ± 0.07

**Table:** 

**Cell lines:**							
**adult human brain-derived neural progenitor cells**
**human glioblastoma cell line**
**primary human glioblastoma cells**

**Table:** 

**Cq >35**	**no mRNA expression**
**Cq = 30-34**	**very low mRNA expression**
**25<Cq ≤ 30**	**low mRNA expression**
**Cq ≤ 25**	**high mRNA expression**

a Cq ± SEM, n=8, for details see Experimental Section. For abbreviations see **Table 2A**.

### 3.5 Sequencing of NPP1 in U87 glioblastoma cells

Cancer cell lines typically show many mutations. To investigate whether NPP1 expressed in the human glioblastoma cell line U87 corresponds to the wildtype sequence or bears mutations, we performed a sequencing analysis based on extraction of RNA (for details see Experimental 2.8). Our data clearly confirmed that the sequence of NPP1 expressed in U87 cells was identical to the wildtype human NPP1 sequence (see **Supplementary Figure 4**).

### 3.6 Studies on membrane preparations of the glioblastoma cell line U87

**Figure 6:**
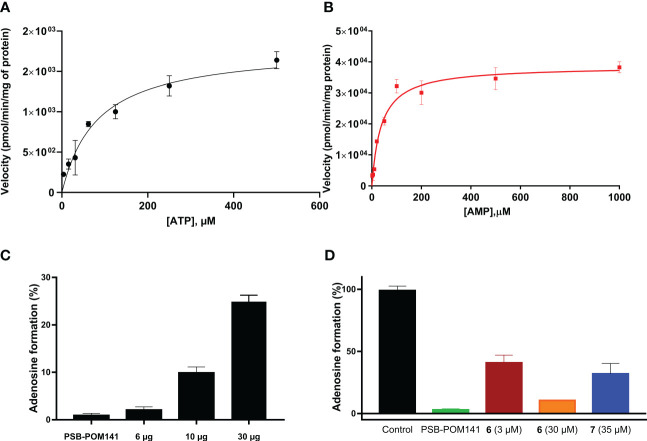
Enzyme kinetics for ATP **(A)** and AMP **(B)** as substrates of ectonucleotidases expressed in U87 cell membrane preparations. **(C)** Enzymatic activity: three different amounts of U87 cell membrane preparations were incubated with 400 µM ATP at 37°C for 30 min, followed by heating to deactivate the enzymes. As a control the known NPP1 inhibitor PSB-POM141 (20 µM) was tested (19). **(D)** Blockade of adenosine formation from ATP by compounds 6 (fondaparinux) and 7 (G9694) in human U87 glioblastoma cell membrane preparations. The potent NPP1 inhibitor PSB-POM141 (20 µM) was added which led to a full blockade of NPP1 activity thereby preventing the formation of AMP and consequently its hydrolysis to adenosine. Control: cell membrane preparation without any test compounds. CE-based methods were employed for the analysis and quantification of the products (for details see Experimental Section).

Next, the selected cell line U87 was biochemically characterized, and kinetic parameters were determined using cell membrane preparations. We investigated the enzymatic activity leading to the formation of adenosine upon incubation with ATP, or AMP, respectively, (**Figure 3C**). For the main natural substrates, K_m_ values of 83 µM (for ATP), and of 38 µM (for AMP) were determined (**Figures 3A, B**). These data are in conformance with data obtained for the isolated enzymes NPP1 and CD73, respectively (12). Next, the inhibitory activity of compounds 7 (G9694) and 6 (fondaparinux) on the formation of adenosine from ATP by U87 cell membrane preparations was studied. Both heparin derivatives inhibited the formation of adenosine in a dose-dependent manner (see **Figure 6**).

### 3.7 NPP1 inhibition and its impact on adenosine formation in U87 glioblastoma cells

As a subsequent step, extracellular ATP degradation by intact U87 cells was analyzed. A CE-based approach was applied to follow ATP degradation and to detect the final formation of adenosine. The cells were pretreated with 20 µM of dipyridamole (DP) to block cellular adenosine uptake by the solute carrier transporters SLC29, better known as equilibrative nucleoside transporters (ENT) (38, 42). Adenosine can be further degraded by adenosine deaminase (ADA) to inosine, a reaction that was observed to take place in U87 cells as indicated by the measured inosine levels (**Supplementary Figure 5A**). The ADA inhibitor *erythro*-9-(2-hydroxy-3-nonyl)adenine (EHNA) was therefore added to prevent adenosine degradation by ADA (43). Combined blockade of cellular adenosine uptake and its conversion to inosine by ADA allowed to confirm the crucial role of NPP1 in extracellular adenosine formation (**Supplementary Figure 5B**). In control experiments, NPP1 activity was inhibited by pretreatment with the potent NPP1 inhibitor PSB-POM-141 (20), which almost completely blocked AMP formation from ATP, and consequently also resulted in a complete blockade of adenosine formation (**Supplementary Figure 5C**).

**Figure 7:**
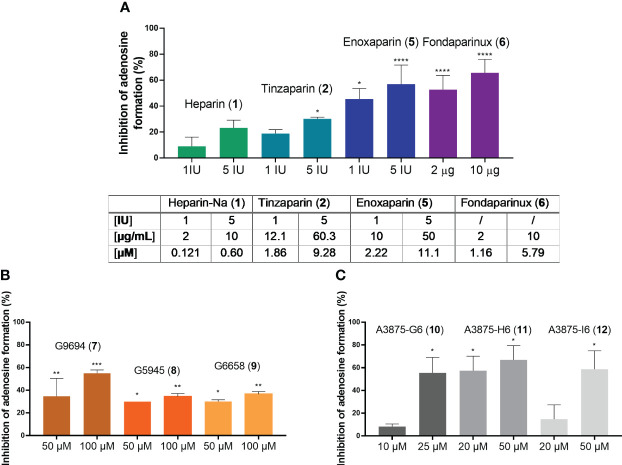
Blockade of extracellular adenosine formation from ATP on human U87 glioblastoma cells **(A)** by compounds 1, 2, 5, and 6 as representatives of commercial heparin derivatives, and **(B, C)** by compounds 7, 8, 9, 10, 11, and 12 as representatives of non-commercial heparin derivatives. The cells were treated with 300 µM of ATP as substrate, and incubated for 3 h in the presence of DP (to prevent cellular uptake of adenosine) and EHNA (to prevent adenosine deamination). Adenosine was quantified by CE-UV and the inhibition calculated and normalized (for details see Experimental Section, **Supplementary Figure 5**). Statistical analysis: one-way ANOVA (normal vs treatment). * p-value ≤ 0.05, ** p-value ≤ 0.01, *** p-value ≤ 0.001, **** p-value ≤ 0.0001.

Heparins were tested in a range of different concentrations. All investigated compounds led to a dose-dependent inhibition of adenosine formation. To be able to relate the tested concentrations to the clinically employed concentrations of these anticoagulants, we adapted them to the therapeutically applied amounts, given in international units (I.U.) for heparins 1, 2 and 3, and in µg for fondaparinux (6). To use a comparable scale and to select a mean therapeutic concentration, as well as a higher one that slightly exceeds the typical therapeutic doses, we selected 1 I.U./mL and 5 I.U./mL for UFH (1), tinzaparin (2) and enoxaparin (5), and 2 µg/mL and 10 µg/mL for fondaparinux (6). The non-commercial heparin derivatives G9694 (7), G5645 (8), G6658 (9) were tested at 50 µM and 100 µM, compounds A3875-H6 (11) and A3875-I6 (12) were studied at 20 and 50 µM, while A3875-G6 (10) was evaluated at 10 and 25 µM. All investigated heparin derivatives were able to inhibit adenosine formation from ATP in U87 glioblastoma cells (**Figure 7**). In fact, UFH (1), and LMWH 2 and 5 inhibited adenosine formation by 30-60% using 5 I.U., while fondaparinux (6) showed even stronger inhibition of adenosine formation, by 60% using 10 µg/mL (~ 6 µM) (**Figure 7A**). These results are comparable to the data obtained on soluble recombinant human NPP1, and on U87 membrane preparations, respectively (**Figure 6D**). They impressively demonstrate the relevance of this mechanism in a cellular system.

The non-commercial heparin derivatives showed a potent, but lower inhibitory activity on the formation of adenosine in U87 tumor cells compared to the commercial derivatives (**Figures 7B, C**). Notably, much higher concentrations were needed to achieve an inhibition in the range of 30 to 50%. Although a structural correlation of this finding appears complicated by the different parameters, a crucial impact of the charge density on inhibitory capacity appears likely, considering the low activities of the desulfated derivatives 8 and 9 (**Figure 7B**). The findings on the size-fractionated derivatives 10-12 (**Figure 7C**), which appear highly active at concentrations of 20 µM and 50 µM, indicate that a smaller heparin fragment is sufficient for NPP1 inhibition, see fondaparinux (6).

**Figure 8:**
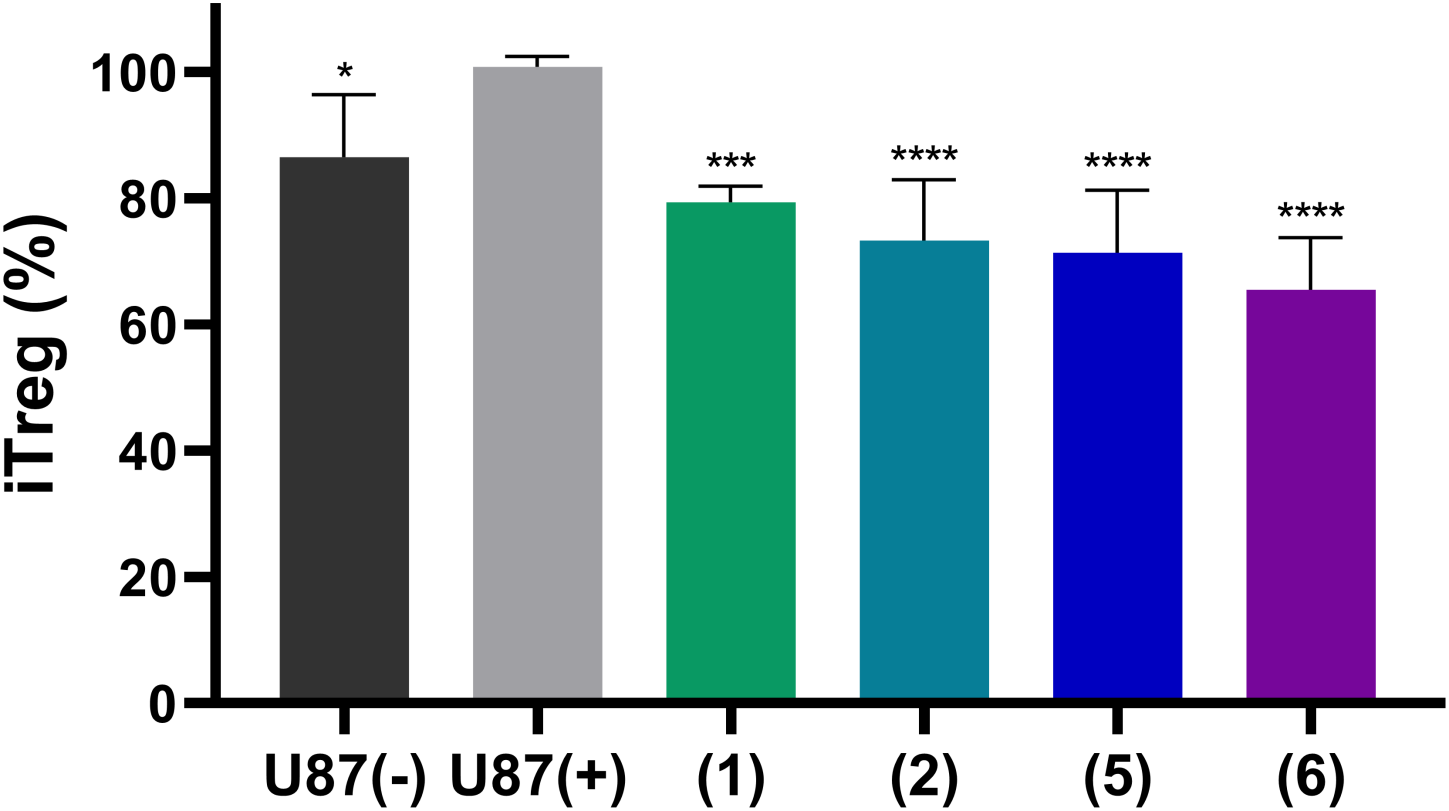
Induction of regulatory T cells from naïve CD4^+^ T cells by stimulation with antibodies and treatment with supernatants of U87 cells. U87 glioblastoma cells were pre-treated with the NPP1 substrate ATP (20 µM) in the presence of dipyridamole to prevent cellular uptake of adenosine, and EHNA to prevent adenosine deamination. The representative commercial heparins UFH (1), tinzaparin (2), enoxaparin (5) as well as the pentasaccharide fondaparinux (6) were added to block extracellular adenosine formation. Percentage of CD4^+^ T cell differentiation in Treg cells and the attenuation by the indicated heparin derivatives is shown. Further details of the flow cytometry analysis areas are shown in **Supplementary Figure 6**. Seven independent experiments were performed. Statistical analysis: one-way ANOVA (normal vs treatment). * p-value ≤ 0.05, *** p-value ≤ 0.001, **** p value ≤ 0.0001.

Finally, we studied whether adenosine accumulation in cellular supernatants will affect immune cell activity, and whether this can be reversed by NPP1 inhibition through heparins. To this end we utilized human CD4^+^ T cells. The differentiation of CD4^+^ cells into an immunosuppressive Treg subset is a crucial pathway of cancer immune evasion. The induction of Treg cells, detected as CD25/FoxP3 positive cells, was analyzed by flow cytometry. First, we measured the impact of supernatants of either untreated U87 cells ‘(U87 (-))’ or those preincubated with 20 µM of the ectonucleotidase substrate ATP ‘(U87 (+))’. The latter solution induced a stronger differentiation of CD4^+^ T cells in Treg cells than the controls, indicating the formation of extracellular adenosine from ATP by the U87 cells. This value was considered as maximum effect, defined as 100% (**Figure 8**). Notably, when the U87 cells preincubated with 20 µM ATP were additionally treated with one of the indicated heparin derivatives, the percentage rate of Treg formation was significantly reduced. All investigated heparins, UFH (1), tinzaparin (2), enoxaparin (5), and particularly fondaparinux (6), reduced Treg formation even below that of the control cells (treated with U87 cell supernatant without ATP addition), indicating that NPP1 expressed in T cells (44) is also inhibited by heparins.

These data clearly confirm the immunosuppressive activity of adenosine formed from ATP by ectonucleotidases in U87 glioblastoma cells, and possibly also in T cells, as well as its reversal by the NPP1-blocking heparins.

## 4 Discussion

All investigated heparin derivatives and analogs exhibited significant inhibitory activity on NPP1 and were found to be NPP1-selective among a large array of investigated ectonucleotidases. Structure-dependent activities were observed, which provide a basis for future optimization, increasing NPP1 inhibition while decreasing or abolishing the compounds’ antithrombotic effect. NPP1 catalyzes the hydrolysis of antiproliferative ATP to produce, in conjunction with the AMP-hydrolyzing ectoenzyme CD73, adenosine, which has immunosuppressive and tumor-promoting properties. Indeed, we observed decreased adenosine production by human U87 glioblastoma cells treated with representative heparin derivatives. Heparins exhibited a significant effect on adenosine formation that was demonstrated to be NPP1-dependent, suggesting a role for immuno-oncological therapies.

Heparin and LMWH are well tolerated and safe anti-thrombotic drugs. UFH is used in a therapeutic range of 8,000-10,000 IU, i.v. or s.c. every 8 h, or 15,000-20,000 IU s.c. every 12 h with plasma concentrations reported to range from 0.2 IU/mL to 15 IU/ml on average (45, 46) and a half-life (T_1/2_) of 1-1.5 h (46). In the present study, compound 1 (UFH) was found to inhibit NPP1 with an IC_50_ value of ~ 4 IU/mL which is in the range of the plasma concentration reached in patients at therapeutic doses. It is worth mentioning that the IC_50_ value of UFH for inhibiting P-selectin is 24.5 µg/mL (47), while at NPP1 the compound is 3-fold more potent (8.26 µg/mL).

LMWHs (e.g., 2, 3, 4 and 5) reach the following Cmax values: Tinzaparin (2) dosed 175 IU/kg body weight s.c. once daily reaches ca. 0.5-1.0 IU/ml (48). Dalteparin (3), dosed once daily with 120 IU/kg s.c., or i.v., respectively, reaches 0.6-2.2 IU/ml (49). Nadroparin (4) applied s.c. in doses of 41 U/kg, or 166 U/kg, respectively, results in maximal concentrations of 0.61-1.34 IU/ml (50). Enoxaparin (5), applied s.c., or initially by i.v. bolus injection, in doses of 30 mg (corresponding to 3000 IU) once daily, or 1 mg/kg (depending on the indication), respectively, reaches levels of 0.2-1.3 IU/ml (51). After several days of therapy mean peak levels of about 1.2 IU are obtained (51). Thus, the average concentrations of these LMWHs appear to be below the levels required for significant NPP1 inhibition.

The therapeutic dosage for fondaparinux (6) is typically 2.5 mg daily, and plasma concentrations have been reported to range from 0.1 to 0.5 mg/L for adult patients on thromboprophylaxis treatment, and from 0.6 to 1.5 mg/L for adults on therapeutic doses, the compound showing a half-life of 17 h (52). These concentrations are also just below the IC_50_ value determined for NPP1 inhibition. However, the negatively charged sulfopolyglycosides may accumulate at the extracellular cell membrane, where NPP1 is located, and local concentrations might actually be higher.

NPP1 inhibition may be beneficial for personalized medicine to treat cancers that show high NPP1 expression. Besides some brain tumors (53), certain liver, thyroid, and head-and neck cancers may be NPP1-positive (54). For example, increasing bone metastasis in breast cancer patients has been associated with high NPP1 expression levels (55). Additional studies in further NPP1-expressing cancer cell lines should be performed. It is, however, unlikely that heparins will induce strong direct antiproliferative effects on cancer cells. Reducing adenosine levels in the microenvironment of cancer cells and tissues by NPP1 inhibition is expected to primarily activate immune cells enabling them to infiltrate and attack cancer tissues (56). Therefore, co-cultures, preferably cancer-immune cell organoids, should be employed (57), followed by *in vivo* studies in suitable animal models.

In humans, NPP1 is predominantly expressed in bone, cartilage, and adipose tissue, but also, for example, in heart, liver, kidney, placenta and testis (14, 54). It is an important source of PP_i_, and its inhibition will not only reduce adenosine, but also PP_i_ concentrations. Since PPi is a key inhibitor of mineralization (58), NPP1 inhibitors might eventually lead to abnormal bone development and soft tissue including blood vessel calcification (59). In fact, chronic application of heparins has been associated with decreased bone stability and soft tissue calcification (60), although conflicting results have been obtained (61). The observed effects might be – at least in part – associated with the potent NPP1-inhibition of heparins. Utilizing heparins with decreased NPP1-inhibitory effects may reduce these side-effects of long-term anticoagulant therapy, e.g. in dialysis patients. In conclusion, we discovered that heparin and its derivatives are potent allosteric inhibitors of the ectonucleotidase NPP1. This mechanism of action might contribute to their clinically observed anti-cancer effects. Based on our results, further studies are warranted investigating the consequences of NPP1 inhibition by heparins *in vivo* on cancers, e.g. in immune-competent mouse models. Combination therapies with anti-cancer drugs should be performed to evaluate potential synergistic effects. In case of positive results, clinical trials applying one of the most potent NPP1-inhibiting heparins may confirm this hypothesis.

## Data availability statement

The following sequencing data have been deposited in the Genbank, accession numbers are given in brackets: SUB12934589 ENPP1_U87_wt (OQ569911) SUB12934589 ENPP1_U87_K173Q (OQ569912).

## Author contributions

CM designed and supervised the study; VL performed experiments on NPP1, NPP4 and U87 membrane preparations. RI, CR, LS, SM, S-YL and VL performed selectivity experiments on various ectonucleotidases. HS and MS performed cell-based experiments under the supervision of GB. HS established and performed the immunological studies and performed with VV cDNA sequencing experiments of U87. AN synthesized and provided non-commercially available synthetic heparins. JP and JS produced and provided NTPDase preparations. BS collected and provided patient-derived cell samples. KS performed qRT-PCR analysis experiments. VL and CM wrote the manuscript with contribution from all coauthors. All authors have given approval to the final version of the manuscript.

## References

[1] Casu B Vlodavsky I Sanderson RD. Non-anticoagulant heparins and inhibition of cancer. Pathophysiol Haemost Thromb (2008) 36(3–4):195–203. doi: 10.1159/000175157 19176992 PMC2768601

[2] Ma SN Mao ZX Wu Y Liang MX Wang DD Chen X. The anti-cancer properties of heparin and its derivatives: a review and prospect. Cell Adhes Migr (2020) 14(1):118–28. doi: 10.1080/19336918.2020.1767489 PMC7513850 32538273

[3] Atallah J Khachfe HH Berro J Assi HI. The use of heparin and heparin-like molecules in cancer treatment: a review. Cancer Treat Res Commun (2020) 24:100192. doi: 10.1016/j.ctarc.2020.100192 32673846

[4] Streiff MB Holmstrom B Angelini D Ashrani A Elshoury A Fanikos J. Cancer-associated venous thromboembolic disease, version 2.2021, nccn clinical practice guidelines in oncology. J Natl Compr Cancer Netw JNCCN (2021) 19(10):1181–201. doi: 10.6004/jnccn.2021.0047 34666313

[5] Khorana AA Mackman N Falanga A Pabinger I Noble S Ageno W. Cancer-associated venous thromboembolism. Nat Rev Dis Primers (2022) 8(1):11. doi: 10.1038/s41572-022-00336-y 35177631

[6] Kakkar AK Levine MN Kadziola Z Lemoine NR Low V Patel HK. Low molecular weight heparin, therapy with dalteparin, and survival in advanced cancer: the fragmin advanced malignancy outcome study (FAMOUS). J Clin Oncol Off J Am Soc Clin Oncol (2004) 22(10):1944–8. doi: 10.1200/JCO.2004.10.002 15143088

[7] Smorenburg SM Van Noorden CJF. The complex effects of heparins on cancer progression and metastasis in experimental studies. Pharmacol Rev (2001) 53(1):93–105. 11171940

[8] Borsig L. Antimetastatic activities of heparins and modified heparins. experimental evidence. Thromb Res (2010) 125:S66–71. doi: 10.1016/S0049-3848(10)70017-7 20434009

[9] Vlodavsky I Singh P Boyango I Gutter-Kapon L Elkin M Sanderson RD. Heparanase: from basic research to therapeutic applications in cancer and inflammation. Drug Resist Updat Rev Comment Antimicrob Anticancer Chemother (2016) 29:54–75. doi: 10.1016/j.drup.2016.10.001 PMC5447241 27912844

[10] Bendas G Borsig L. Cancer cell adhesion and metastasis: selectins, integrins, and the inhibitory potential of heparins. Int J Cell Biol (2012) 2012:676731. doi: 10.1155/2012/676731 22505933 PMC3296185

[11] Lopez V Schäkel L Schuh HJM Schmidt MS Mirza S Renn C. Sulfated polysaccharides from macroalgae are potent dual inhibitors of human ATP-hydrolyzing ectonucleotidases NPP1 and CD39. Mar Drugs (2021) 19(2):51. doi: 10.3390/md19020051 33499103 PMC7911304

[12] Zimmermann H. History of ectonucleotidases and their role in purinergic signaling. Biochem Pharmacol (2021) 187:114322. doi: 10.1016/j.bcp.2020.114322 33161020

[13] Barbeau X Mathieu P Paquin JF Lagüe P. Characterization of the structure, dynamics and allosteric pathways of human NPP1 in its free form and substrate-bound complex from molecular modeling. Mol Biosyst (2017) 13(6):1058–69. doi: 10.1039/c7mb00095b 28474020

[14] Lee S-Y Müller CE. Nucleotide pyrophosphatase/phosphodiesterase 1 (npp1) and its inhibitors. MedChemComm (2017) 8(5):823–40. doi: 10.1039/C7MD00015D PMC6072468 30108800

[15] Namasivayam V Lee S-Y Müller CE. The promiscuous ectonucleotidase npp1: molecular insights into substrate binding and hydrolysis. Biochim Biophys Acta - Gen Subj (2017) 1861(3):603–14. doi: 10.1016/j.bbagen.2016.12.019 28011303

[16] Onyedibe KI Wang M Sintim HO. ENPP1, an old enzyme with new functions, and small molecule inhibitors–a STING in the tale of ENPP1. Molecules (2019) 24(22):4192. doi: 10.3390/molecules24224192 31752288 PMC6891441

[17] Kato K Nishimasu H Oikawa D Hirano S Hirano H Kasuya G. Structural insights into CGAMP degradation by ecto-nucleotide pyrophosphatase phosphodiesterase 1. Nat Commun (2018) 9(1):4424. doi: 10.1038/s41467-018-06922-7 30356045 PMC6200793

[18] Carozza JA Brown JA Böhnert V Fernandez D AlSaif Y Mardjuki RE. Structure-aided development of small-molecule inhibitors of enpp1, the extracellular phosphodiesterase of the immunotransmitter CGAMP. Cell Chem Biol (2020) 27(11):1347–1358.e5. doi: 10.1016/j.chembiol.2020.07.007 32726585 PMC7680421

[19] Naggi A Casu B Perez M Torri G Cassinelli G Penco S. Modulation of the heparanase-inhibiting activity of heparin through selective desulfation, graded n-acetylation, and glycol splitting. J Biol Chem (2005) 280(13):12103–13. doi: 10.1074/jbc.M414217200 15647251

[20] Lee S-Y Fiene A Li W Hanck T Brylev KA Fedorov VE. Polyoxometalates-potent and selective ecto-nucleotidase inhibitors. Biochem Pharmacol (2015) 93(2):171–81. doi: 10.1016/j.bcp.2014.11.002 25449596

[21] Lee S-Y Sarkar S Bhattarai S Namasivayam V De Jonghe S Stephan H. Substrate-dependence of competitive nucleotide pyrophosphatase/phosphodiesterase1 (NPP1) inhibitors. Front Pharmacol (2017) 8:54. doi: 10.3389/fphar.2017.00054 28261095 PMC5309242

[22] Gorelik A Randriamihaja A Illes K Nagar B. Structural basis for nucleotide recognition by the ectoenzyme CD203c. FEBS J (2018) 285(13):2481–94. doi: 10.1111/febs.14489 29717535

[23] Gorelik A Randriamihaja A Illes K Nagar B. A key tyrosine substitution restricts nucleotide hydrolysis by the ectoenzyme NPP5. FEBS J (2017) 5:1–9. doi: 10.1111/febs.14266 28898552

[24] Lopez V Lee S-Y Stephan H Müller CE. Recombinant expression of ecto-nucleotide pyrophosphatase/phosphodiesterase 4 (npp4) and development of a luminescence-based assay to identify inhibitors. Anal Biochem (2020) 603:113774. doi: 10.1016/j.ab.2020.113774 32445636

[25] Schäkel L Mirza S Winzer R Lopez V Idris R Al-Hroub H. Protein kinase inhibitor ceritinib blocks ectonucleotidase CD39 - a promising target for cancer immunotherapy. J Immunother Cancer (2022) 10(8):e004660. doi: 10.1136/jitc-2022-004660 35981785 PMC9394215

[26] Kaczmarek E Koziak K Sévigny J Siegel JB Anrather J Beaudoin AR. Identification and characterization of CD39 vascular ATP diphosphohydrolase. J Biol Chem (1996) 271:33116–22. doi: 10.1074/jbc.271.51.33116 8955160

[27] Knowles AF Chiang WC. Enzymatic and transcriptional regulation of human ecto-ATPase/E-NTPDase 2. Arch Biochem Biophys (2003) 418(2):217–27. doi: 10.1016/j.abb.2003.08.007 14522593

[28] Smith TM Kirley TL. Cloning, sequencing, and expression of a human brain ecto-apyrase related to both the ecto-ATPases and CD39 ecto-apyrases1. Biochim Biophys Acta - Protein Struct Mol Enzymol (1998) 1386:65–78. doi: 10.1016/s0167-4838(98)00063-6 9675246

[29] Fausther M Lecka J Kukulski F Lévesque SA Pelletier J Zimmermann H. Cloning, purification, and identification of the liver canalicular ecto-ATPase as NTPDase8. Am J Physiol Gastrointest Liver Physiol (2007) 292(3):G785–95. doi: 10.1152/ajpgi.00293 PMC3952495 17095758

[30] Kukulski F Lévesque SA Lavoie EG Lecka J Bigonnesse F Knowles AF. Comparative hydrolysis of P2 receptor agonists by NTPDases 1, 2, 3 and 8. Purinergic Signal (2005) 1(2):193–204. doi: 10.1007/s11302-005-6217-x 18404504 PMC2096530

[31] Lévesque SA Lavoie EG Lecka J Bigonnesse F Sévigny J. Specificity of the ecto-ATPase inhibitor ARL 67156 on human and mouse ectonucleotidases. Br J Pharmacol (2007) 152(1):141–50. doi: 10.1038/sj.bjp.0707361 PMC1978278 17603550

[32] Freundlieb M Zimmermann H Müller CE. A New, sensitive ecto-5′-nucleotidase assay for compound screening. Anal Biochem (2014) 446:53–8. doi: 10.1016/j.ab.2013.10.012 24144488

[33] Junker A Renn C Dobelmann C Namasivayam V Jain S Losenkova K. Structure-activity relationship of purine and pyrimidine nucleotides as ecto-5’-nucleotidase (CD73) inhibitors. J Med Chem (2019) 62(7):3677–95. doi: 10.1021/acs.jmedchem.9b00164 PMC6526958 30895781

[34] Sievers F Wilm A Dineen D Gibson TJ Karplus K Li W. Fast, scalable generation of high-quality protein multiple sequence alignments using clustal omega. Mol Syst Biol (2011) 7(1):539. doi: 10.1038/msb.2011.75 21988835 PMC3261699

[35] Bradford MM. A rapid and sensitive method for the quantitation of microgram quantities of protein utilizing the principle of protein-dye binding. Anal Biochem (1976) 72(1–2):248–54. doi: 10.1016/0003-2697(76)90527-3 942051

[36] Iqbal J Vollmayer P Braun N Zimmermann H Müller CE. A Capillary electrophoresis method for the characterization of ecto-nucleoside triphosphate diphosphohydrolases (NTPDases) and the analysis of inhibitors by in-capillary enzymatic micro reaction. Purinergic Signal (2005) 1(4):349–58. doi: 10.1007/s11302-005-8076-x PMC2096555 18404519

[37] Qurishi R Kaulich M Müller CE. Fast, efficient capillary electrophoresis method for measuring nucleotide degradation and metabolism. J Chromatogr A (2002) 952(1–2):275–81. doi: 10.1016/s0021-9673(02)00095-x 12064539

[38] Pastor-Anglada M Pérez-Torras S. Who is who in adenosine transport. Front Pharmacol (2018) 9:627. doi: 10.3389/fphar.2018.00627 29962948 PMC6010718

[39] Poppe D Doerr J Schneider M Wilkens R Steinbeck JA Ladewig J. Genome editing in neuroepithelial stem cells to generate human neurons with high adenosine-releasing capacity. Stem Cells Transl Med (2018) 7(6):477–86. doi: 10.1002/sctm.16-0272 PMC5980162 29589874

[40] Kaulich M Qurishi R Müller CE. Extracellular metabolism of nucleotides in neuroblastoma x glioma ng108-15 cells determined by capillary electrophoresis. Cell Mol Neurobiol (2003) 23(3):349–64. doi: 10.1023/a:1023640721630 PMC11530159 12825832

[41] Gómez-Villafuertes R Pintor J Miras-Portugal MT Gualix J. Ectonucleotide pyrophosphatase/phosphodiesterase activity in neuro-2a neuroblastoma cells: changes in expression associated with neuronal differentiation. J Neurochem (2014) 131(3):290–302. doi: 10.1111/jnc.12794 24947519

[42] Zimmerman MA Tak E Ehrentraut SF Kaplan M Giebler A Weng T. Equilibrative nucleoside transporter (ent)-1-dependent elevation of extracellular adenosine protects the liver during ischemia and reperfusion. Hepatol Baltim md (2013) 58(5):1766–78. doi: 10.1002/hep.26505 PMC3795856 23703920

[43] Yegutkin GG. Enzymes involved in metabolism of extracellular nucleotides and nucleosides: functional implications and measurement of activities. Crit Rev Biochem Mol Biol (2014) 49(6):473–97. doi: 10.3109/10409238.2014.953627 25418535

[44] Stefan C Jansen S Bollen M. Modulation of purinergic signaling by NPP-type ectophosphodiesterases. Purinergic Signal (2006) 2(2):361. doi: 10.1007/s11302-005-5303-4 18404476 PMC2254485

[45] Wahr JA Yun JH Yang VC Lee LM Fu B Meyerhoff ME. A new method of measuring heparin levels in whole blood by protamine titration using a heparin-responsive electrochemical sensor. J Cardiothorac Vasc Anesth (1996) 10(4):447–50. doi: 10.1016/S1053-0770(05)80002-3 8776635

[46] Russel DH Garcia DA Burnett AE. Heparin and LMW heparin: dosing and adverse effects. Available at: https://www.uptodate.com/contents/heparin-and-lmw-heparin-dosing-and-adverse-effects#H40.

[47] Gomes AM Kozlowski EO Borsig L Teixeira FCOB Vlodavsky I Pavão MSG. Antitumor properties of a new non-anticoagulant heparin analog from the mollusk nodipecten nodosus: effect on p-selectin, heparanase, metastasis and cellular recruitment. Glycobiology (2015) 25(4):386–93. doi: 10.1093/glycob/cwu119 25367817

[48] Kuhle S Massicotte P Dinyari M Vegh P Mitchell D Marzinotto V. Dose-finding and pharmacokinetics of therapeutic doses of tinzaparin in pediatric patients with thromboembolic events. Thromb Haemost (2005) 94(6):1164–71. doi: 10.1160/TH05-03-0215 16411388

[49] Available at: https://www.pfizermedicalinformation.ca/en-ca/fragmin/action-and-clinical-pharmacology.

[50] Jaspers TCC Meijer CE Vleming LJ Franssen CFM Diepstraten J Lukens MV. AM J Physiol Gastrointest LiverPhysiol (2007) 292(3):G785-95. doi: 10.1007/s40262-022-01162-x

[51] Available at: https://pdf.hres.ca/dpd_pm/00047708.PDF.

[52] Depasse F Gerotziafaz GT Busson J Van Dreden P Samama MM. Assessment of three chromogenic and one clotting assays for the measurement of synthetic pentasaccharide fondaparinux (Arixtra®) anti-xa activity. J Thromb Haemost (2004) 2(2):346–8. doi: 10.1111/j.1538-7933.2004.0584a.x 14996008

[53] Aerts I Martin JJ De Deyn PP Van Ginniken C Van Ostade X Kockx M. The expression of ecto-nucleotide pyrophosphatase/phosphodiesterase 1 (E-NPP1) is correlated with astrocytic tumor grade. Clin Neurol Neurosurg (2011) 113(3):224–9. doi: 10.1016/j.clineuro.2010.11.018 21195542

[54] Available at: https://www.proteinatlas.org/ENSG00000197594-ENPP1/pathology.

[55] Lau WM Doucet M Stadel R Huang D Weber KL Kominsky SL. Enpp1: a potential facilitator of breast cancer bone metastasis. PloS One (2013) 8(7):e66752. doi: 10.1371/journal.pone.0066752 23861746 PMC3702501

[56] Boison D Yegutkin GG. Adenosine metabolism: emerging concepts for cancer therapy. Cancer Cell (2019) 36(6):582–96. doi: 10.1016/j.ccell.2019.10.007 PMC7224341 31821783

[57] Visalakshan RM Lowrey MK Sousa MGC Helms HR Samiea A Schutt CE. Opportunities and challenges to engineer 3D models of tumor-adaptive immune interactions. Front Immunol (2023) 14:1162905. doi: 10.3389/fimmu.2023.1162905 37081897 PMC10110941

[58] Hajjawi MO MacRae VE Huesa C Boyde A Millán JL Arnett TR. Mineralisation of collagen rich soft tissues and osteocyte lacunae in Enpp1(-/-) mice. Bone (2014) 69:139–47. doi: 10.1016/j.bone.2014.09.016 PMC4228085 25260930

[59] Orriss IR. Extracellular pyrophosphate: the body's "water softener". Bone (2020) 134:115243. doi: 10.1016/j.bone.2020.115243 31954851

[60] Meng Y Zhang H Li Y Li Q Zuo L. Effects of unfractionated heparin on renal osteodystrophy and vascular calcification in chronic kidney disease rats. Bone (2014) 58:168–76. doi: 10.1016/j.bone.2013.10.010 24145307

[61] Niu Q Yang S Gan L Zhao H Zuo L. Different type and dosage of heparin were not associated with the progression of coronary artery calcification in haemodialysis patients. Nephrol (Carlton) (2020) 25(7):551–8. doi: 10.1111/nep.13632 PMC7317585 31339604

[62] Lopez V. Identification and pharmacological characterization of nucleotide pyrophosphatase/phosphodiesterase-1 (NPP1) and-4 (NPP4) inhibitors. Bonn, Germany: Doctoral Dissertation (2022). Available at: https://bonndoc.ulb.uni-bonn.de/xmlui/handle/20.500.11811/10115.

