# Supplementary material for: Heparins are potent inhibitors of ectonucleotide pyrophosphatase/phospho-diesterase-1 (NPP1) – a promising target for the immunotherapy of cancer
Source: Front Immunol. 2023 Aug 29;14:1173634. doi: 10.3389/fimmu.2023.1173634 (PMC10497752; doi:10.3389/fimmu.2023.1173634)
Supplement: Supplementary file 1 [file DataSheet_1.pdf]

Supplementary Material

Heparins are potent inhibitors of ectonucleotide pyrophosphatase/phosphodiesterase-1 (NPP1) – a promising target for the immunotherapy of cancer

Vittoria Lopez,[1,2,&] H.J. Maximilian Schuh,[3,&] Salahuddin Mirza,[1,2] Victoria J. Vaaßen,[1,2] Michael S. Schmidt,[3] Katharina Sylvester,[1,2] Riham Mohammed Idris,[1,2] Christian Renn,[1,2] Laura Schäkel,[1,2] Julie Pelletier,[4,5] Jean Sévigny,[4,5] Annamaria Naggi,[6] Björn Scheffler,[7] Sang-Yong Lee,[1,2] Gerd Bendas,[3]* and Christa E. Müller[1,2]*

[&]equal contribution

*** Correspondence:**

christa.mueller@uni-bonn.de

gbendas@uni-bonn.de

**Table of content**
**Supplementary Figure 1.** Initial screening of selected commercial heparins on selected ectonucleotidases
**Supplementary Figure 2.** Inhibition curves of investigated compounds on NPP1
**Supplementary Figure 3.** Efficacy of primers used for mRNA gene analysis
**Supplementary Figure 4.** Sequence analysis of NPP1 in U87 cells
**Supplementary Figure 5.** Capillary electrophoresis-based detection of adenosine formation in supernatants of ATP-treated U87 cells and its inhibition by heparins
**Supplementary Figure 6.** Gating strategy to identify the subset of induced regulatory T cells

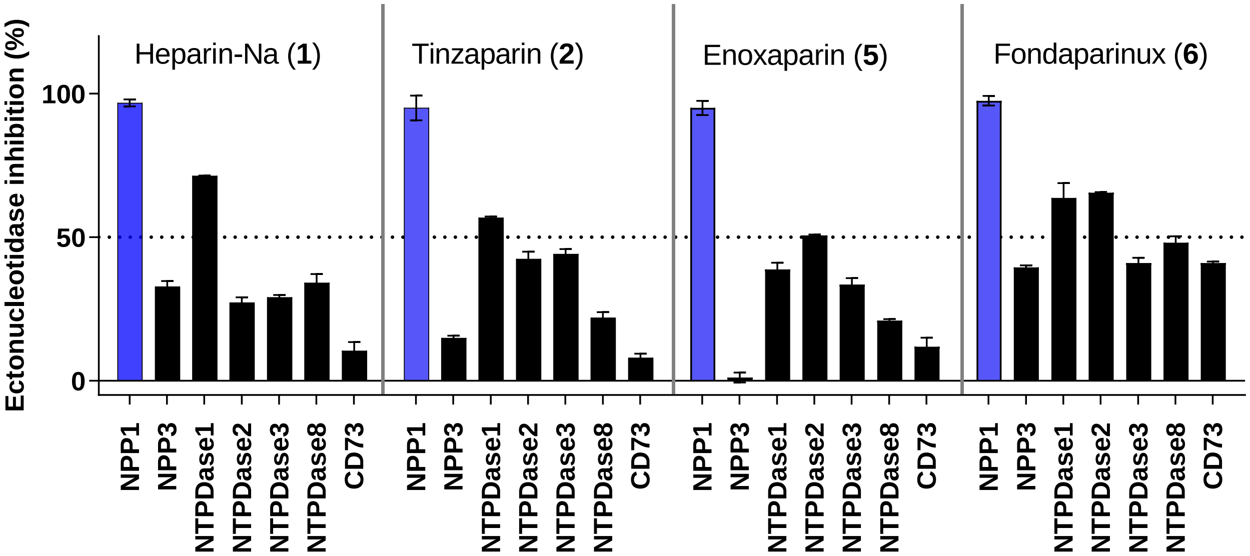

Supplementary Figure 1. Initial screening of selected commercial heparins on selected ectonucleotidases. The compounds were tested at the following concentrations: compounds **1** and **2** at 63 IU/ml, compound **5** at 250 µg/ml (55 µM), compound **6** was tested at 50 µg/mL (30 µM). For detailed information about the assays, see material and methods.

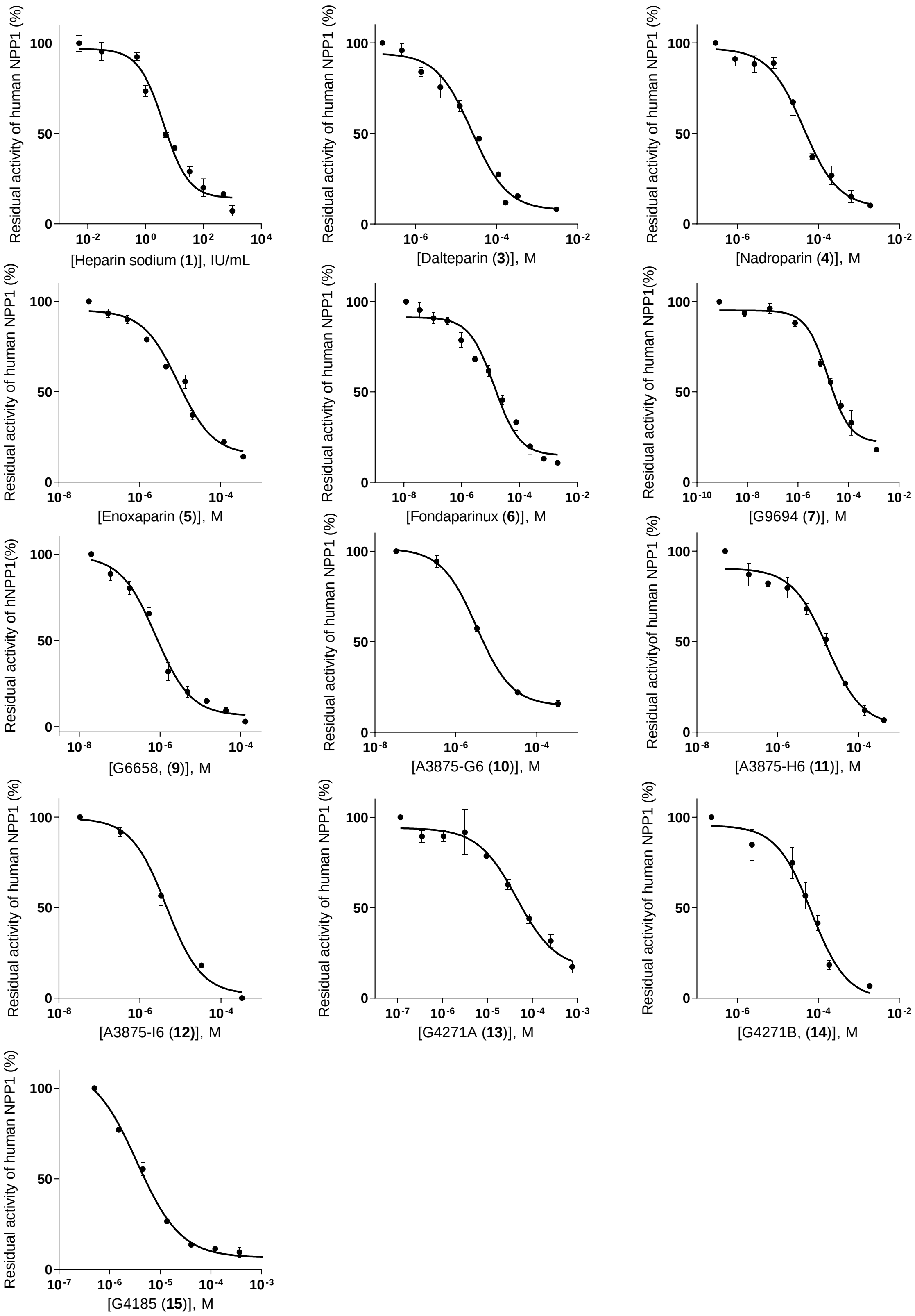

Supplementary Figure 2. Inhibition curves of investigated compounds on NPP1. IC_{50} values of (**8**) and (**2**) were extrapolated from % inhibition. For details, see experimental section.

A) hENTPD1

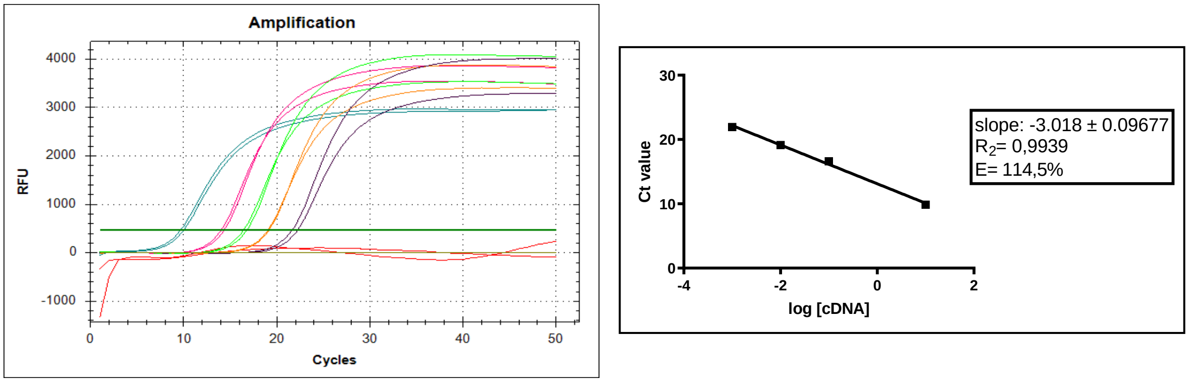

B) hENTPD2

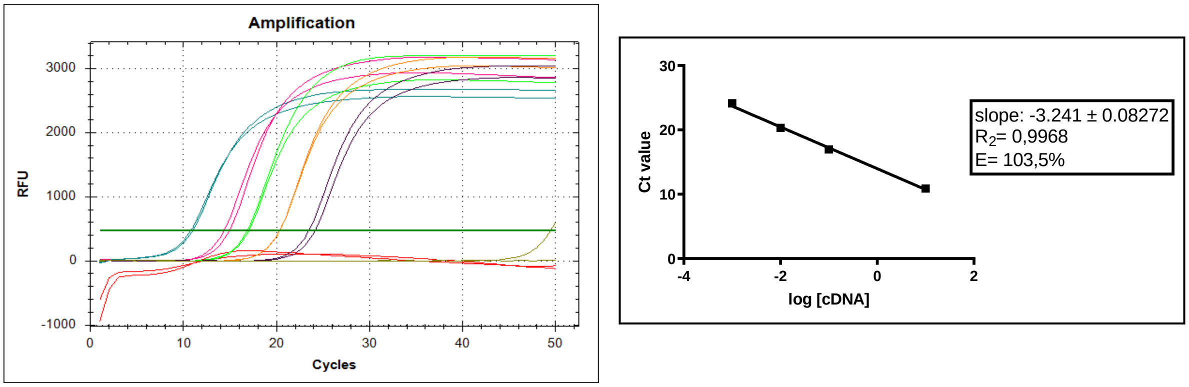

C) hENTPD3

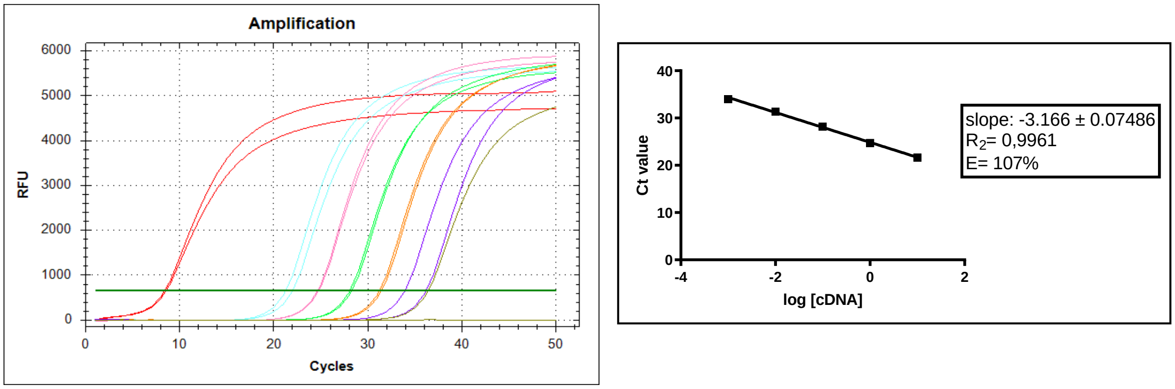

D) hENTPD8

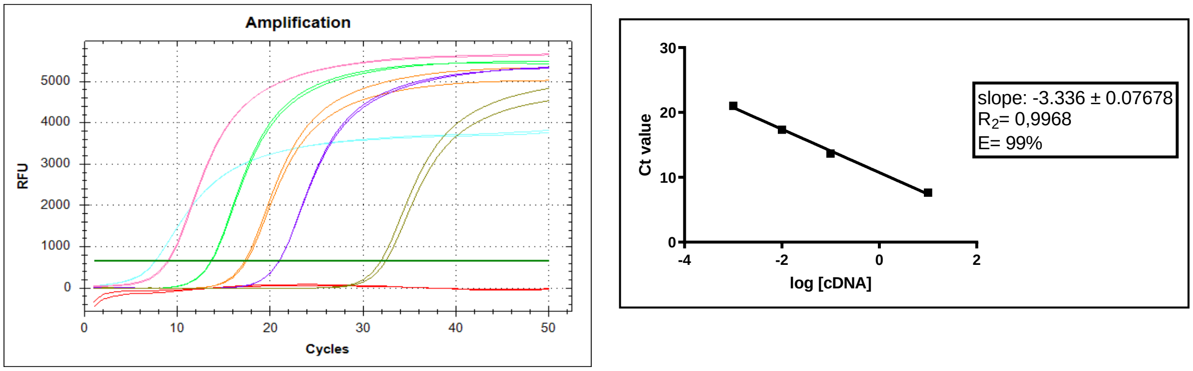

E) hENPP1

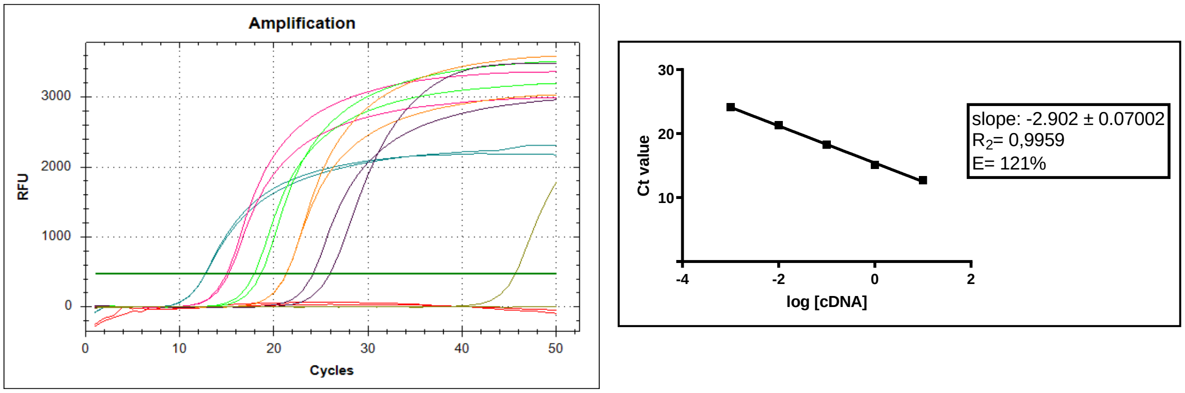

F) hENPP2

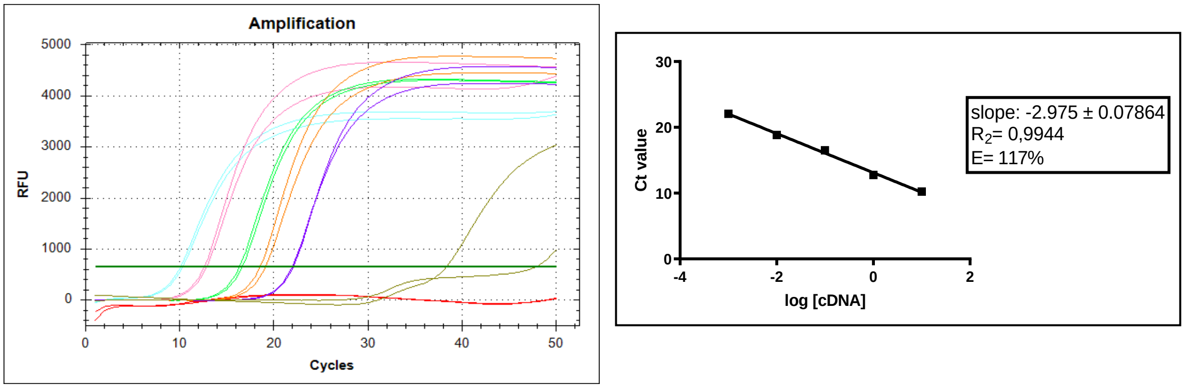

G) hENPP3

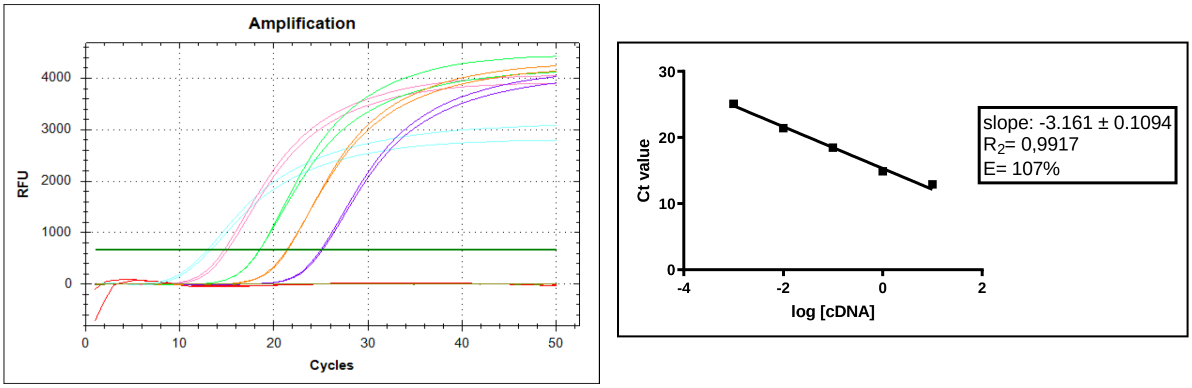

H) hNT5E

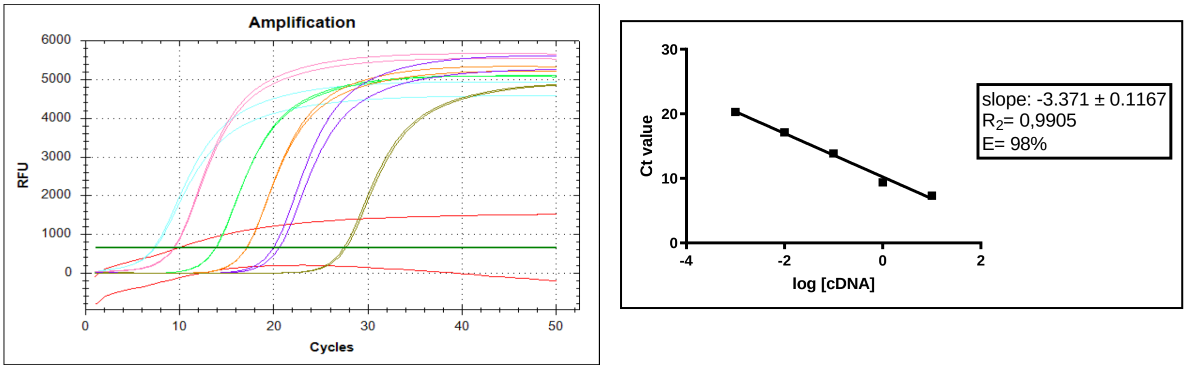

Supplementary Figure 3. Efficacy of primers used for mRNA analysis. The colors in the amplification cycles graphics represent replicate of different concentration of specific primer and on the right side its related standard curves with calculated slope and efficacy (E). Optimal efficacy range is 90-110 %.

hENPPs: human ectonucleotide pyrophosphatase; hENTPDs: human ectonucleoside triphosphate diphosphohydrolases; hNT5E: human ecto-5'-nucleotidase gene; RFU: relative fluorescence units; Ct: cycle threshold; E: efficacy.

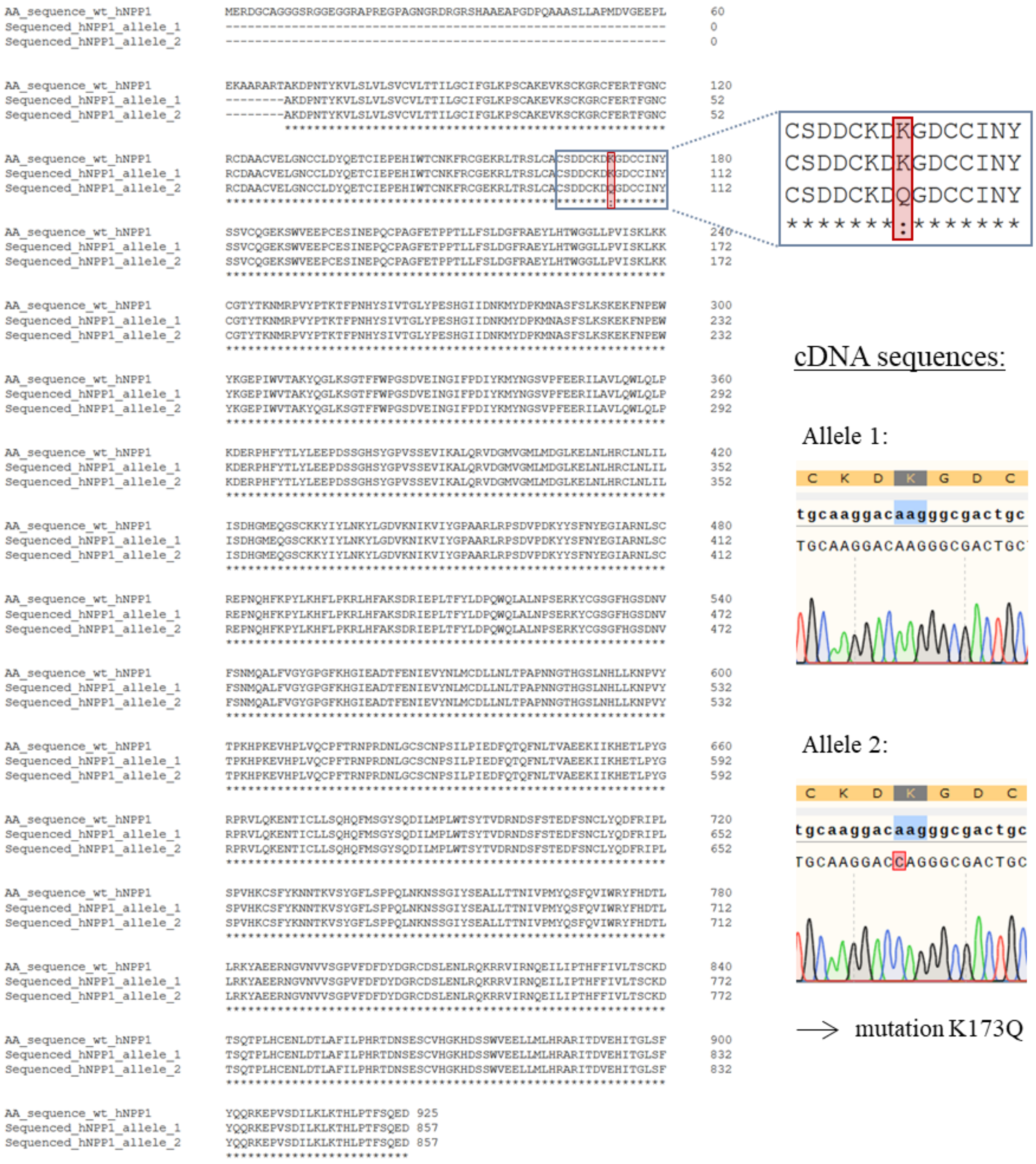

Supplementary Figure 4. NPP1 complementary DNA (cDNA) sequencing results of U87 cells. Amino acid sequence alignment of wildtype (wt) NPP1 with the transcription of both identified alleles. A known single nucleotide polymorphism (SNP) was identified in the base sequence of one allele (A > C) resulting in mutation of the amino acid lysine in position 173 to glutamine (K173Q). This mutation is also referred to as K121Q (Lys121Gln) if counted from the ATG start codon after 156 bp. It slightly decreases the enzymatic activity to 66 % producing lower amounts of PP_i and is linked to several metabolic diseases. The other allele was identified as the wt sequence. Due to the high GC-content at the beginning of the NPP1 gene, sequencing primers were designed to align downstream (amino acid 69) resulting in a shorter sequence as the wt NPP1. The missing sequence does not contain any known SNPs.

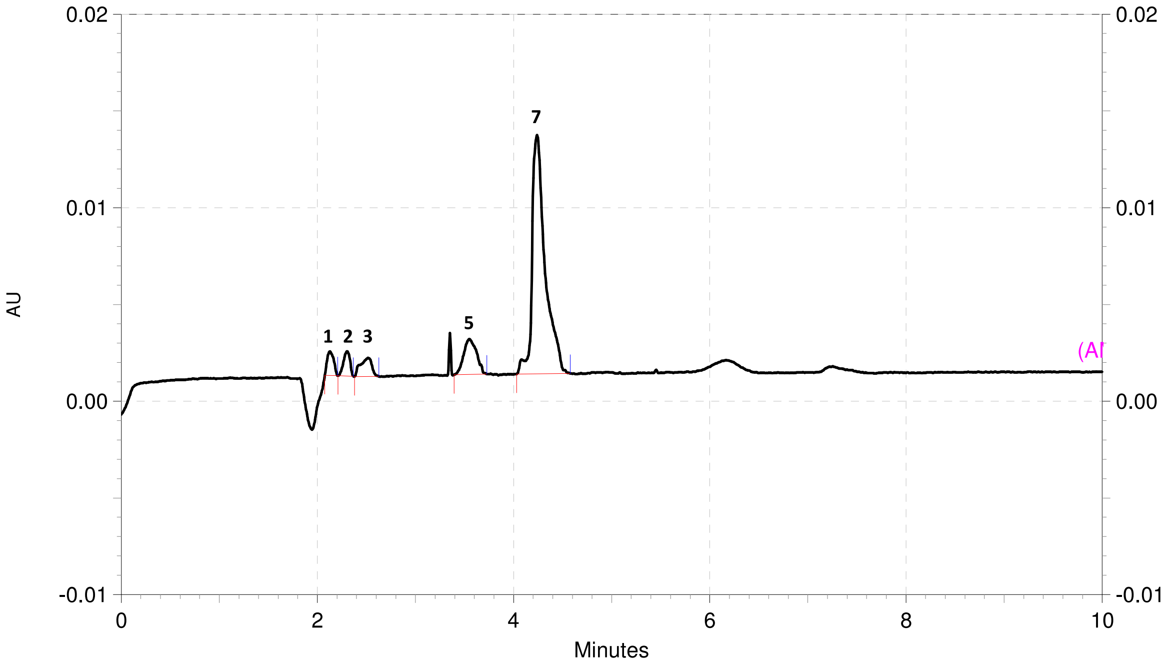

Supplementary Figure 5A. Electropherogram of a supernatant of U87 cells treated solely with 300 µM ATP. The sample was detected after 3 hours of cell incubation with ATP 300 µM and DP 20 µM without inhibition of ectonucleotidase activity. 1 = Uridine, 2 = Inosine, 3 = Adenosine, 5 = AMP, 7 = ATP

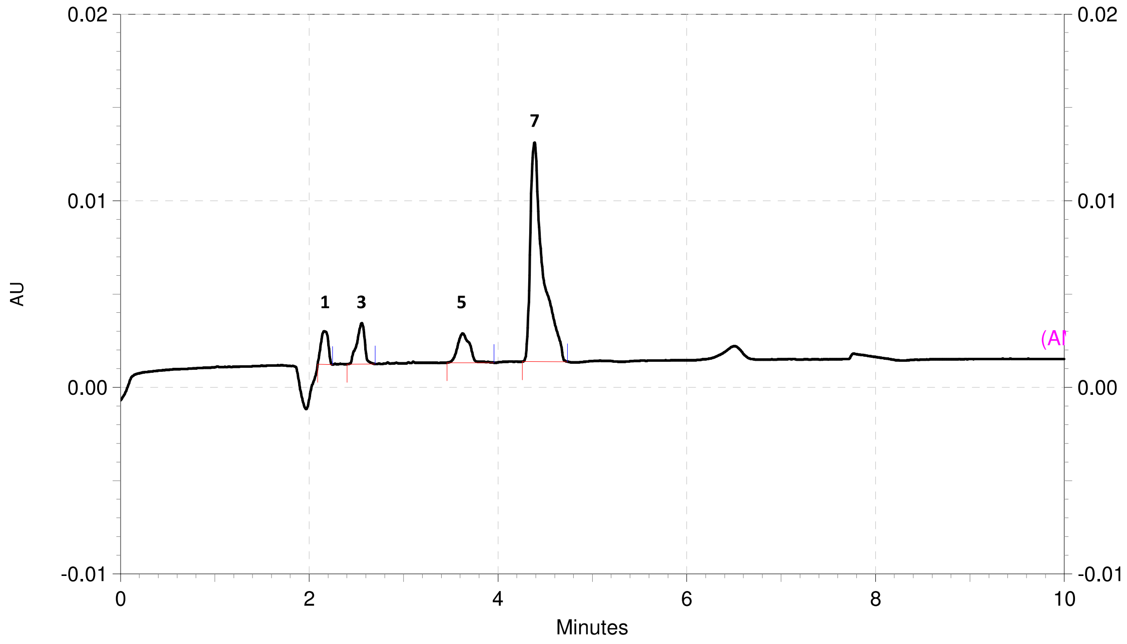

Supplementary Figure 5B.Electropherogram of a U87 cell supernatant after treating the cell with ATP 300 µM and blocking adenosine cellular uptake and extracellular conversion into inosine by DP

20 µM and ADA inhibitor EHNA 1µM, respectively. 1 = Uridine, 3 = Adenosine, 5 = AMP, 7 = ATP

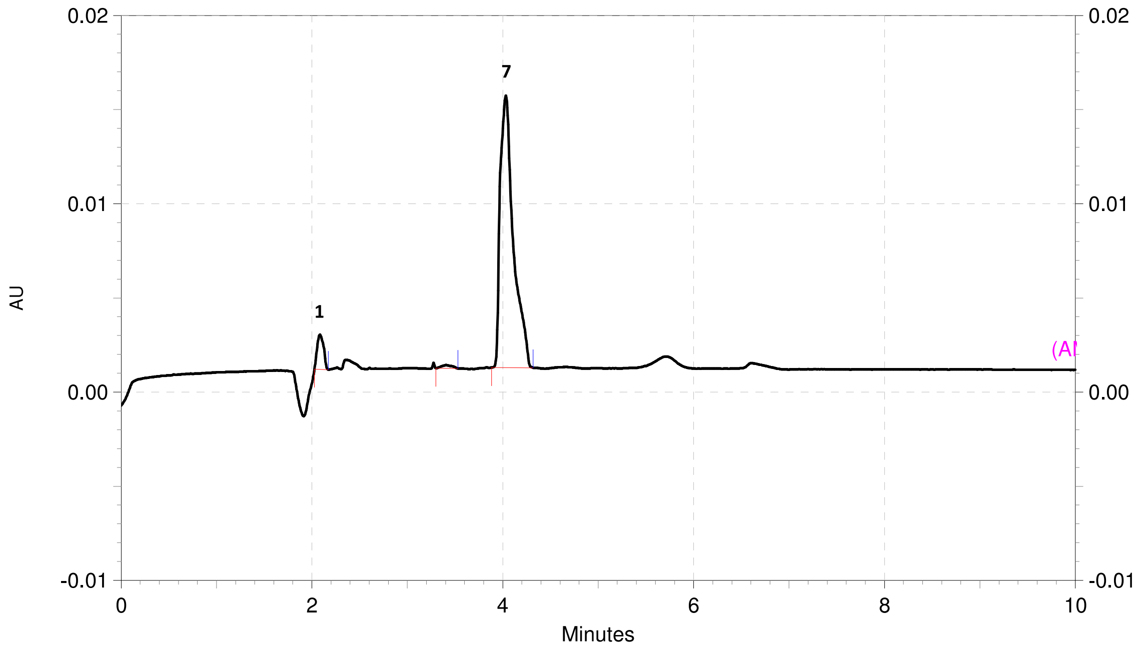

Supplementary Figure 5C. Electropherogram of a U87 cell supernatant pretreated with ATP 300 µM, DP 20 µM, EHNA 1 µM and NPP1 inhibitor PSB-POM-141 at 10 µM. The measurement was performed after 3 hours of incubation with ATP. 1 = Uridine, 7 = ATP

Calculation

The percentage of inhibition of adenosine formation in U87 cell supernatants by heparin derivatives was calculated from the conversion rate as follows:

$$Conversion\ Rate\ [\%] = \frac{AUC\ (ADO)}{[AUC\ (ADO) + AUC\ (AMP) + AUC\ (ATP)]} * 100$$

Taking the AUC of the indicated components in each electropherogram and relating to the maximum inhibition by PSB-POM-141 at 10 µM (0% conversion) and 100 % conversion in the control experiment (shown in Supplementary figure 5B).

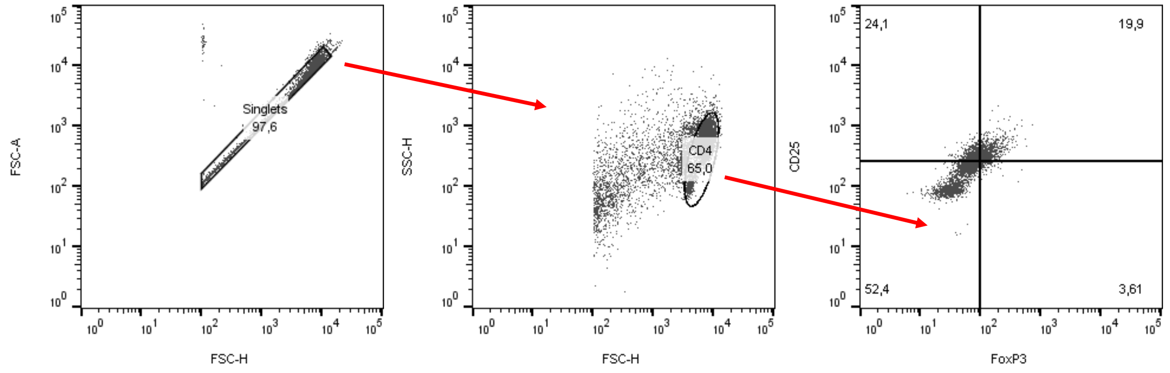

Supplementary Figure 6A. Gating strategy to identify the subset of induced regulatory T cells. A doublet discrimination was performed to gate the singlets by applying FSC area against height. By setting up another gate with the parameters SSC vs. FSC, the $CD4^+$ cells are separated from remaining cell debris. Subsequently, the percentage of CD25 and FoxP3 positive cells was determined, which is representative for the induced regulatory T cells.

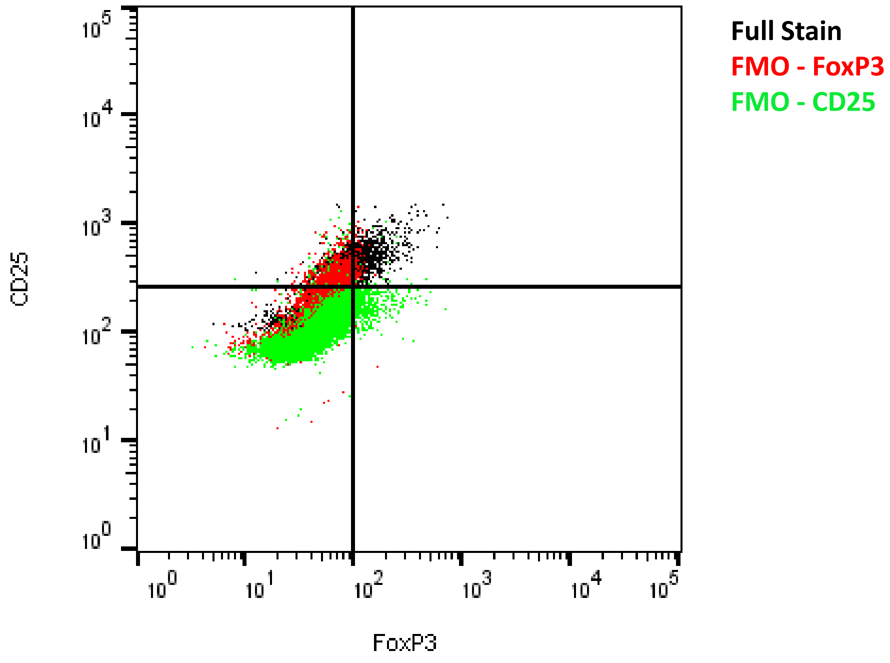

Supplementary Figure 6B. FMO controls of CD25 (green) and FoxP3 (red) plotted together with a sample of activated $CD4^+$ T cells treated with supernatant of cell line U87 (black)

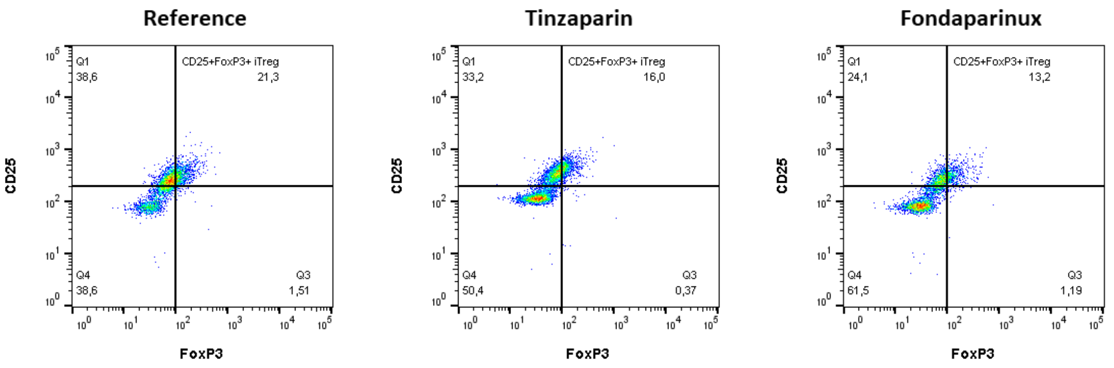

Supplementary Figure 6C. Representative dot plots of the flow cytometric analysis after 96 h display the induction of Treg cells (left) and the attenuated induction by the treatment with tinzaparin and fondaparinux, respectively.
